# Structural basis for promiscuous action of monoterpenes on TRP channels

**DOI:** 10.1038/s42003-021-01776-0

**Published:** 2021-03-05

**Authors:** Thi Hong Dung Nguyen, Satoru G. Itoh, Hisashi Okumura, Makoto Tominaga

**Affiliations:** 1 Department of Physiological Sciences, SOKENDAI, Okazaki, Japan; 2 Division of Cell Signaling, National Institute for Physiological Sciences, Okazaki, Japan; 3 Thermal Biology Group, Exploratory Research Center on Life and Living Systems (ExCELLS), Okazaki, Japan; 4 Biomolecular Dynamics Simulation Group, ExCELLS, Okazaki, Japan; 5 Research Center for Computational Science, Institute for Molecular Science, Okazaki, Japan; 6 Department of Structural Molecular Science, SOKENDAI, Okazaki, Japan

**Keywords:** Ion channels in the nervous system, Molecular conformation

## Abstract

Monoterpenes are major constituents of plant-derived essential oils and have long been widely used for therapeutic and cosmetic applications. The monoterpenes menthol and camphor are agonists or antagonists for several TRP channels such as TRPM8, TRPV1, TRPV3 and TRPA1. However, which regions within TRPV1 and TRPV3 confer sensitivity to monoterpenes or other synthesized chemicals such as 2-APB are unclear. In this study we identified conserved arginine and glycine residues in the linker between S4 and S5 that are related to the action of these chemicals and validated these findings in molecular dynamics simulations. The involvement of these amino acids differed between TRPV3 and TRPV1 for chemical-induced and heat-evoked activation. These findings provide the basis for characterization of physiological function and biophysical properties of ion channels.

## Abstract (web summary)

Thi Hong Dung Nguyen et al. identify which regions within TRP channels TRPV1 and TRPV3 confer their sensitivity to monoterpenes that are major constituents of plant-derived essential oils. This study provides insights into how different TRP channels respond to chemical or heat stimuli.

## Introduction

Organisms use sensors such as transient receptor potential (TRP) ion channels to adapt to environmental changes. Members of the TRP ion channel family play important roles as polymodal sensors to detect and respond to changes in temperature, pH, voltage, osmolarity, and exogenous molecules involved in taste, smell, and pheromone responses^1^. Channels in this family include TRP vanilloid 1 (TRPV1), TRP vanilloid 3 (TRPV3), and TRP melastatin 8 (TRPM8) that are all crucial for sensing temperature and natural compounds^2,3^. TRPV1 is physiologically important for thermal and chemical nociception in sensory neurons and is activated by capsaicin, toxins, pH, and temperature in the noxious range (>42 °C). The third member of the TRPV subfamily, TRPV3, is also a heat sensor. TRPV3 is expressed in various tissues and organs, but the most intensive expression is in skin epithelial cells. TRPV3 is believed to be responsible for sensation of warm temperature ranging from 35 to 39 °C^4^, but has also been reported to be initially activated by noxious heat (>50 °C) and sensitized to activation in response to warm temperature^5^. Chemical agonists of TRPV3 include spice extracts that contain monoterpenes such as menthol, camphor, and synthetic agents (including 2-aminoethoxy diphenylborate, 2-APB) and unsaturated fatty acids^6^. Whereas many TRPV subfamily members act as heat sensors, the TRPM8 channel is known to be involved in sensation induced by cool temperatures (<22–27 °C) and menthol^3,7^. TRMP8 is highly expressed in sensory neurons of trigeminal and dorsal root ganglia, and in the prostate epithelium^8^. PIP_2_ regulates TRM8 activity and PIP_2_ depletion can desensitize the channel^9–13^.

The six TRP subfamilies (TRPC, TRPV, TRPM, TRPA, TRPP, and TRPML) all have a tetrameric assembly consisting of six transmembrane secondary structures (S1–S6) with intracellular N and C termini. Each subunit has a re-entrant loop with a pore loop (P) comprising 1–2 small pore helices (H1/H2) that is located between S5 and S6 (S5–P–S6). The S1–S4 and S5–P–S6 are connected by the S4–S5 linker. The structures of several TRP channels were recently extensively clarified by cryo-EM single-particle analysis^14–17^. The combination of cryo-EM microscopy with nanodisc technology allowed the efficient determination of the location of annular and regulatory lipids^9,15,18^. Lipids in cellular membranes are important regulators that can functionally modulate many TRP channels by direct or specific interactions, especially in the case of phosphatidylinositides (PIP_2_) and cholesterol. Gao et al.^15^ reported that a binding pocket in the S4–S5 linker region for vanilloid ligands is shared with that for PIP_2_. Tightly bound PIP_2_ works as a co-factor that stabilizes TRPV1 in its resting state by serving as a competitive vanilloid antagonist and a negative allosteric modulator^14,15^. In a recent study, Singh et al. observed two non-protein densities, presumably representing lipids, in each subunit of the TRPV3 tetramer. The pockets for lipids are located in the intracellular half of S1–S4 domains and C terminus of the TRP domain, which are analogous to the pocket for putative lipids in other TRPV channels^16^. Lipid location and function were also clarified in recent studies of the TRPM8 structure^9,18^. Yin et al. (2019) reported that for TRPM8 PIP_2_ can effectively control conformational transitions associated with gating and enhance the potency of agonist binding^9,17^.

Menthol, a natural non-reactive cooling compound that has three asymmetric carbon atoms and a molecular formula of C_10_H_20_O, is widely used in oral hygiene products, cosmetics, pesticides, and pharmaceuticals, and is also used as a flavoring agent in foods. The major form of menthol found in nature is (−)-menthol (*l*-menthol), which is frequently studied since it has better cooling properties than other isomers^19,20^. Menthol is a well-known TRPM8 activator and is required for cool thermosensation in vivo^3,7^. Menthol can also activate TRPV3 and has bimodal effects on *mouse* TRPA1 (refs. ^21,22^). Moreover, several amino acids were shown to be involved in the menthol binding in *mouse* TRPM8 (Y745, R842, and Y1005)^23^ and human TRPA1 (S873 and T874)^22^. We previously reported that menthol inhibits capsaicin-activated human TRPV1 activity^24^, indicating that it shows promiscuous actions toward several TRP channels. However, no consensus has been reached about menthol-binding sites, especially for TRPV3.

Other structurally related monoterpenes such as camphor, carvacrol, and 1,8-cineol can also regulate several TRP channels in different ways. For example, camphor activates mammalian TRPV1 and TRPV3, but inhibits *rat* TRPA1 (ref. ^21^). Interestingly, some studies indicated that these natural compounds can insert into biological membranes where they can cause significant alterations in numerous physico-chemical characteristics of lipid bilayers^25–28^. A central question arises about whether the effects of these small and hydrophobic compounds are mediated directly, via direct interaction with ion channels integrated in the membrane, and/or indirectly through alterations in the physico-chemical characteristics of lipid membranes that have indirect effects on channel function.

Here we have characterized the pharmacological effects of menthol and other structurally related monoterpenes on several TRP channels including TRPV1, TRPV3, and TRPM8. We show an agonistic effect of menthol on TRPV1. We also identified several amino acids in the S4–S5 linker that are involved in the agonistic effect of menthol on *rat* TRPV1 (R557K and G563S) and *mouse* TRPV3 (R567K and G573S).

## Results

### Confirmation of mutations that specifically affect menthol responses in TRPM8

We made histidine substitutions at Y745 in the S1 transmembrane segment and R842 in the S4 transmembrane segment of *mouse* TRPM8 (mTRPM8; Supplementary Figs. 1 and  2a); these amino acids are putative binding sites for menthol on TRPM8 (refs. ^10,22^). We then used a whole-cell patch-clamp method to test the agonistic effects of menthol (Supplementary Fig. 2b) on these mutants expressed in HEK293T cells. Consistent with previous reports, menthol (500 μM)-evoked currents for mTRPM8 Y745H and R842H mutants were much smaller than those of wild type (WT) mTRPM8 (Supplementary Fig. 2c, d), while their cold sensitivity was preserved.

### Involvement of S4–S5 region residues R567 and G573 in agonist responses by mTRPV3

**Fig. 1:**
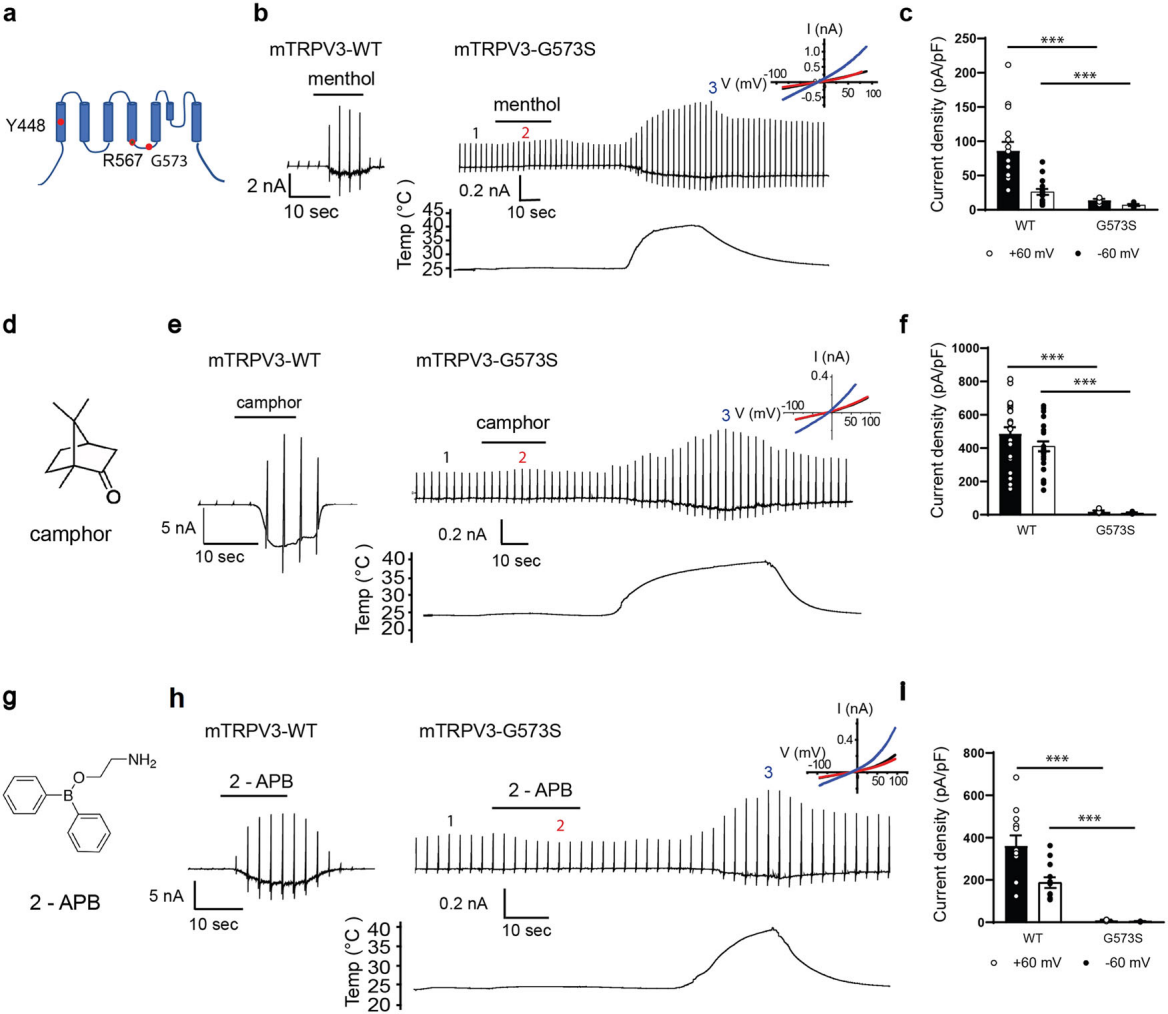
Effect of TRPV3-G573S mutation on the agonistic action of menthol, camphor, and 2-APB. **a** Location of Y448 (S1), R567, and G573S (S4–S5 linker) in *mouse* TRPV3 (mTRPV3). **b** Representative current traces for HEK293T cells expressing WT or mTRPV3-G573S in response to menthol (3 mM) followed by heat stimulation. **c** Comparison of menthol-activated current densities in HEK293T cells expressing WT or mTRPV3-G573S at ±60 mV (WT: 86.4 ± 12.7 pA/pF at +60 and 26.2 ± 4.3 pA/pF at −60 mV, *n* = 16; G573S: 14.0 ± 1.9 pA/pF at +60 mV and 6.9 ± 1.5 pA/pF at −60mV, *n* = 5). **d** Structure of camphor. **e** Representative current traces for WT or mTRPV3-G573S in response to camphor (10 mM) followed by heat stimulation. **f** Comparison of camphor-activated current densities in HEK293T cells expressing WT or mTRPV3-G573S at ±60 mV (WT: 466.0 ± 43.5 pA/pF at +60 mV and 410.8 ± 29.6 pA/pF at −60mV, *n* = 26; G573S: 18.6 ± 6.7 pA/pF at +60 mV and 9.6 ± 3.9 pA/pF, *n* = 5). **g** Structure of 2-APB. **h** Representative current traces for WT or mTRPV3-G573S in response to 2-APB (300 μM) followed by heat stimulation. **i** Comparison of 2-APB-activated current densities in HEK293T cells expressing WT or mTRPV3-G573S at ±60 mV (WT: 361.5 ± 48.8 pA/pF at +60 mV and 187.7 ± 25.3 pA/pF at −60mV, *n* = 12; G573S: 10.7 ± 1.5 pA/pF at +60 mV and 5.1 ± 1.0 pA/pF, *n* = 5). Insets in **b**, **e**, **h** indicate current–voltage curves obtained at the different time points (1, 2, 3) shown in each trace. Holding potentials were −60 mV with ramp-pulses (−100 to +100 mV, 300 ms) applied every 3 s. Data represent means ± SEM. Statistical analysis was performed by two-sample *t*-test, ***p < 0.001.

We hypothesized that amino acids associated with the effects of menthol on TRPV3 and TRPM8 would be similar and found three candidate residues, tyrosine (Y) in S1 and arginine (R) and glycine (G) in the S4–S5 linker (Fig. 1a and Supplementary Fig. 1). In this study, we focused mainly on the shared residues in the S4–S5 linker domain since the glycine residue in TRPM8 corresponding to G573 in mTRPV3 is not known to be part of the menthol-binding site and the S4–S5 linker is known to have broad involvement in several functions of thermosensitive TRP channels. We found that Y448 in mTRPV3 is not involved in menthol-evoked mTRPV3 activation (Supplementary Fig. 3a). Camphor and 2-APB sensitivities were not changed for mTRPV3-Y448H (Supplementary Fig. 3b, c). However, unlike WT mTRPV3, menthol did not activate mTRPV3-G573S, whereas heat-evoked activation was still observed (Fig. 1b, c), indicating that G573 is involved in menthol-evoked activation of mTRPV3. This mutant also lost sensitivity to camphor and 2-APB, but was activated by heat (Fig. 1d–i), consistent with a previous report^29^. The importance of glycine in the S4–S5 linker domain in chemical activation is consistent with the recent result showing heat-evoked activation of TRPV3 wherein temperature-mediated opening of TRPV3 is induced by conformational changes in the extracellular region of the pore domain that then promote channel opening with involvement of the S4–S5 linker domain^30^.

**Fig. 2:**
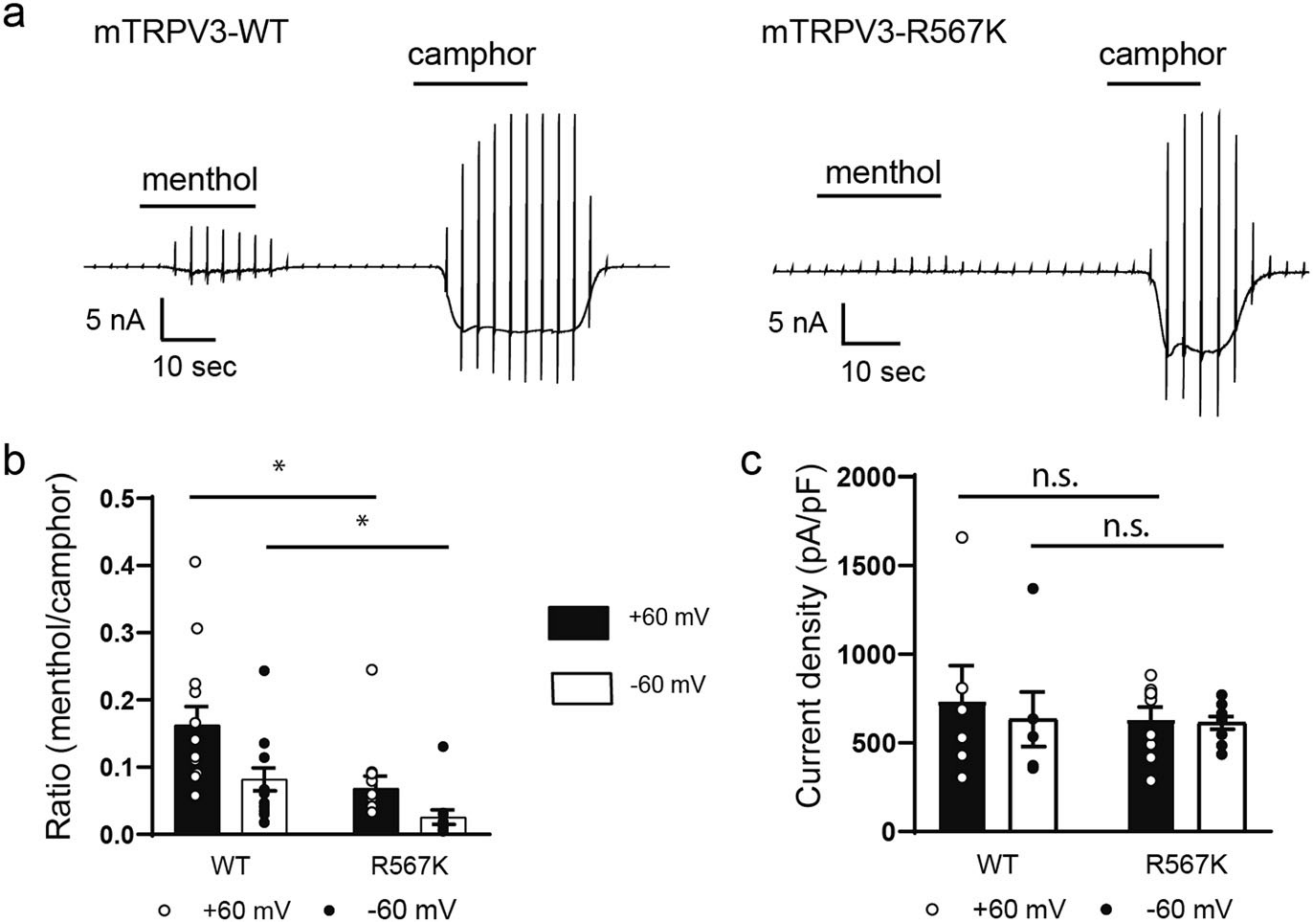
Agonistic effect of menthol, but not camphor, involves R567 in *mouse* TRPV3. **a** Representative current traces for HEK293T cells expressing wild type (WT) or R567K mutant stimulated with menthol (3 mM) followed by camphor (10 mM). **b** Comparison of the normalized current densities (*I*_menthol_/*I*_camphor_) at ±60 mV (WT: 0.16 ± 0.03 at +60 mV and 0.08 ± 0.02 at −60 mV, *n* = 13; R567K: 0.07 ± 0.02 at +60 mV and 0.02 ± 0.01 at −60mV, *n* = 11). **c** Comparison of camphor-activated current densities for HEK293T cells expressing WT or mTRPV3-R567K at ±60 mV (WT: 736.5 ± 198.8 pA/pF at +60 mV and 633.5 ± 153.6 pA/pF at −60 mV, *n* = 6; R567K: 634.9 ± 68.2 pA/pF at +60 mV and 613.6 ± 35.7 pA/pF at −60 mV, *n* = 9). Holding potentials were −60 mV with ramp-pulses (−100 to +100 mV, 300 ms) every 3 s. Data represent means ± SEM. Statistical analysis was performed by two-sample *t*-test, *p < 0.05.

Next, we examined the possible involvement of R567 in menthol-evoked activation of TRPV3. Menthol-activated currents were very small in mTRPV3-R567K (Fig. 2a, b), whereas the magnitude of camphor-evoked currents was similar between WT and mTRPV3-R567K (Fig. 2c), which is in contrast to the phenotype seen for mTRPV3-G573S. Similar results were obtained when the application sequence was changed (Supplementary Fig. 4a, b). The sensitivity of WT and mTRPV3-R567K to 2-APB and camphor was also similar (Supplementary Fig. 4c, d). These data indicated that G573 and R567 are differently involved in the chemical responses of mTRPV3 wherein G573 is involved in responses to menthol, camphor, and 2-APB, while R567 is involved in menthol, but not in camphor and 2-APB sensitivity.

Given the critical location of G573 and R567 at the S4–S5 linker and S4, mutations in this region could produce structural changes that affect channel gating. Structural changes at these sites could also contribute to the observed effect of a ligand beyond that seen for ligand binding alone. To examine this possibility, we analyzed mTRPV3-F569H that carries a mutation that lies in close proximity to the tip of the S4–S5 linker. We found that current sizes in response to menthol and camphor were similar between WT and F569H (Supplementary Fig. 5), indicating that F569 is not involved in the activation of mTRPV3 by monoterpenes.

### Involvement of S4/S4–S5 region residues R557 and G563 in the agonistic effect of menthol on TRPV1

**Fig. 3:**
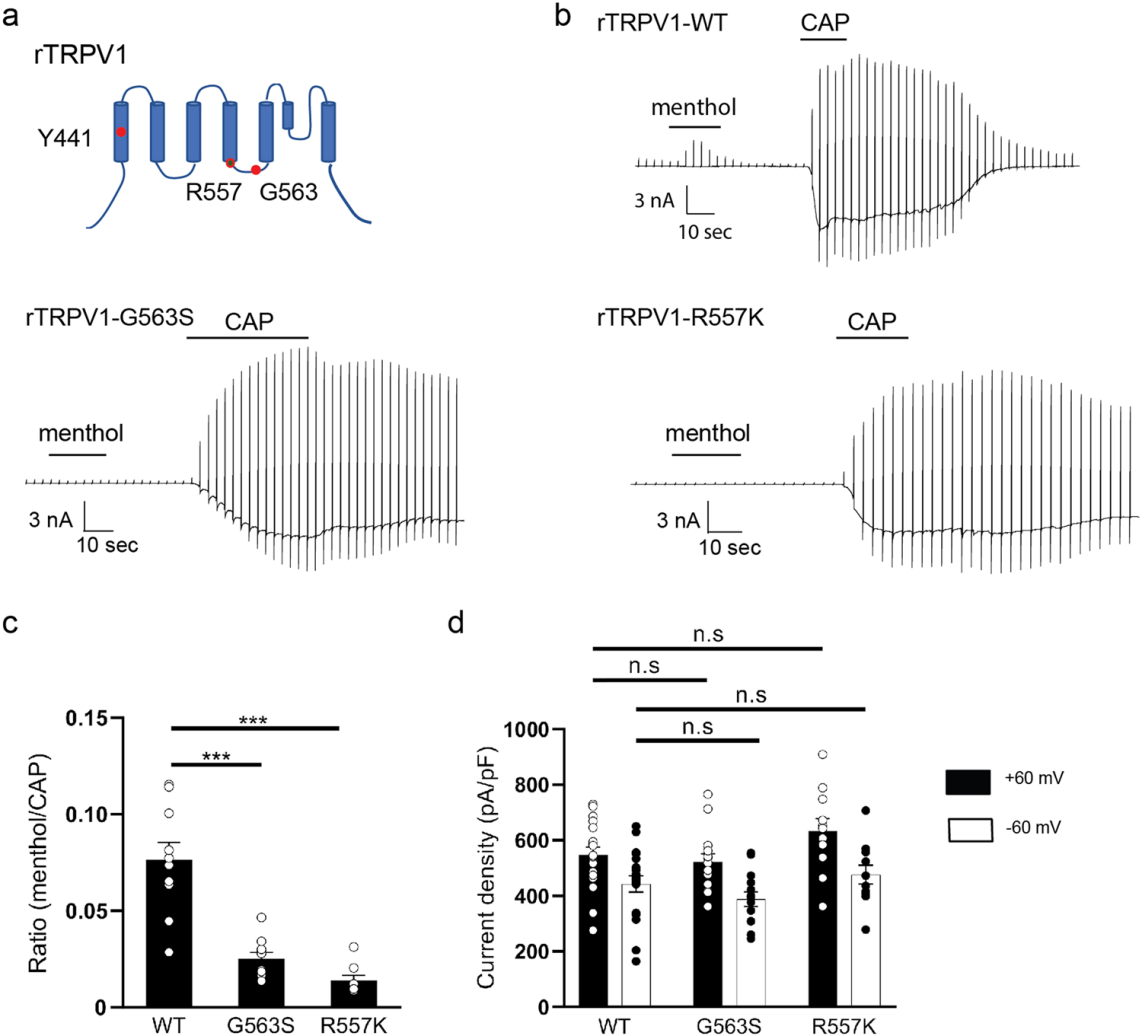
Effect of *rat* TRPV1 mutations G563S and R557K on the agonistic action of menthol. **a** Location of residues Y441 (S1), R557 (bottom of S4), and G563 (S4–S5 linker) in *rat* TRPV1 (rTRPV1). **b** Representative current traces for HEK293T cells expressing WT, G563S, or R557K rTRPV1 in response to menthol (3 mM) followed by capsaicin (CAP, 1 μM) stimulation. **c** Comparison of normalized current densities (*I*_menthol_/*I*_CAP_) for WT, rTRPV1-G563S, or rTRPV1-R557K expressed by HEK293T cells at +60 mV (WT: 0.08 ± 0.01, *n* = 11; G563S: 0.03 ± 0.003, *n* = 10; R557K: 0.01 ± 0.002, *n* = 9). **d** Comparison of CAP-activated currents densities for WT, rTRPV1-G563S, or rTRPV1-R557K expressed in HEK293T cells at ±60 mV (WT: 547 ± 26.7 pA/pF at +60 mV and 442 ± 29.2 pA/pF at −60 mV, *n* = 22; G563S: 521 ± 3.7 pA/pF at +60 mV and 388.1 ± 26.4 pA/pF at −60 mV, *n* = 14; R557K: 633.5 ± 3.3 pA/pF at +60 mV and 476.4 ± 33.8 pA/pF at −60 mV, *n* = 11). Holding potentials were −60 mV with ramp-pulses (−100 to +100 mV, 300 ms) applied every 3 s. Data represent means ± SEM. Statistical analysis was performed using two-sample *t*-test, ***p < 0.001.

Naturally occurring TRP ligands show some degree of promiscuity. Notably, menthol is not only a TRPM8 agonist, but can also activate mammalian TRPV3 (refs. ^10,31^) and TRPA1 (ref. ^32^), suggesting that the mechanism associated with the response to menthol is conserved across several TRP channels. Indeed, we previously reported that hTRPV1 currents activated by capsaicin were inhibited by menthol with IC_50_ values of 1.2 ± 0.2 mM^24^, whereas mTRPM8 activation by menthol has EC_50_ values ranging from 4 to 80 μM^3^. Therefore, we first attempted to examine the agonistic effect of high concentrations of menthol on TRPV1 since both the tyrosine in S1 and arginine and glycine in the S4/S4–S5 linker of rTRPV1 are conserved (Fig. 3a). We did not examine Y441 since capsaicin sensitivity was altered in rTRPV1-Y441H (Supplementary Fig. 6b) and the importance of this aromatic cluster is underscored by previous studies demonstrating that replacement of Y441 by small non-aromatic residues resulted in non-functional channels^33,34^.

**Fig. 4:**
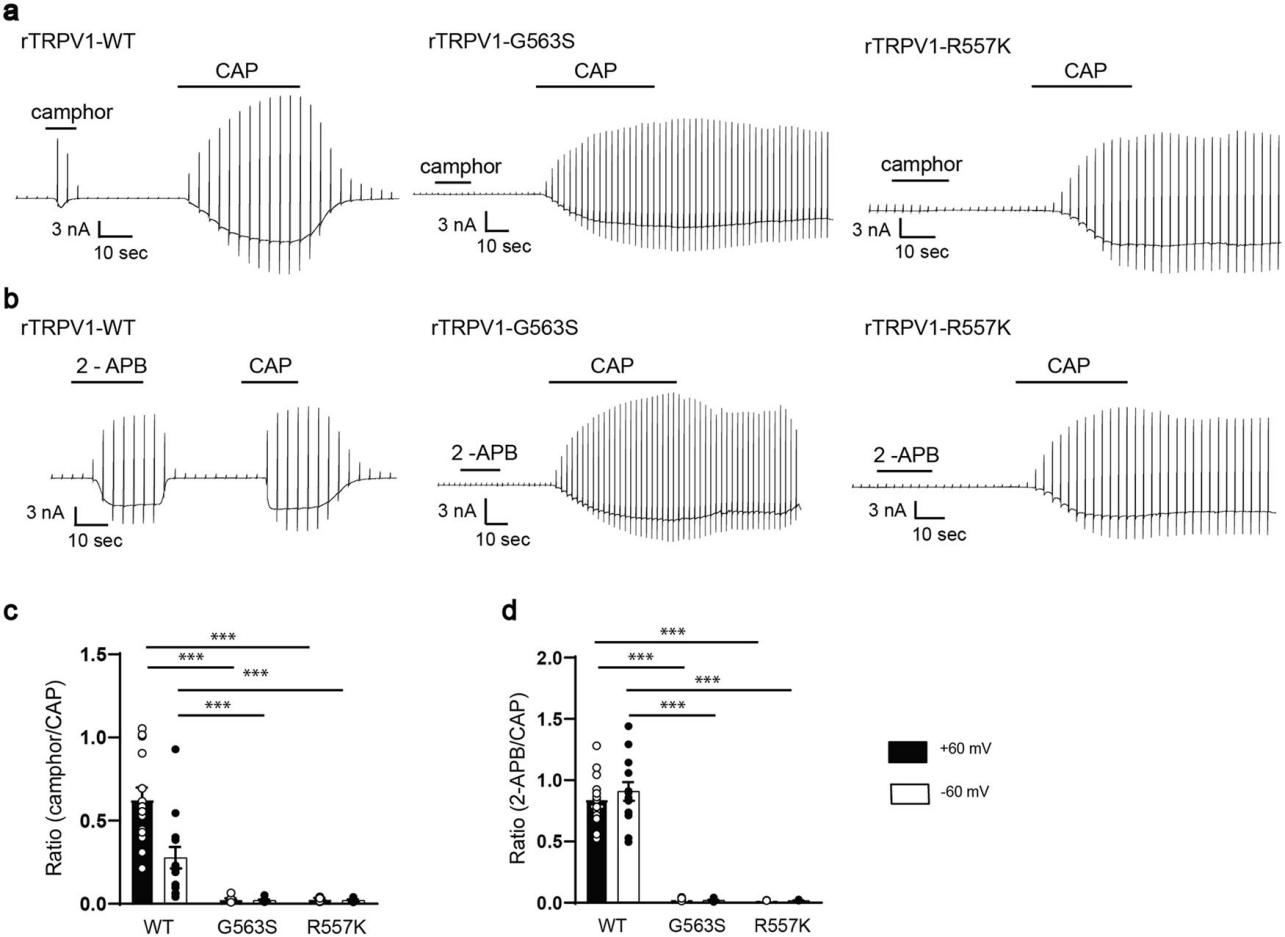
Effect of *rat* TRPV1 mutations G563S and R557K on agonistic action of camphor and 2-APB. **a** Representative current traces for HEK293T cells expressing WT, rTRPV1-G563S, or rTRPV1-R557K stimulated with camphor (10 mM) followed by CAP (1 μM). **b** Representative current traces for HEK293T cells expressing WT, rTRPV1-G563S, or rTRPV1-R557K stimulated with 2-APB (300 μM) followed by CAP (1 μM). **c** Comparison of the normalized current densities (*I*_camphor_/*I*_CAP_) for WT, rTRPV1-G563S, or rTRPV1-R557K expressed in HEK293T cells at ±60 mV (*I*_camphor_/*I*_CAP_ for WT: 0.63 ± 0.07 at +60 mV and 0.28 ± 0.06 at −60 mV, *n* = 14; G563S: 0.03 ± 0.01 at +60 mV and 0.02 ± 0.01 at −60 mV, *n* = 8; R557K: 0.03 ± 0.01 at +60 mV and 0.02 ± 0.01 at −60 mV, *n* = 5). **d** Comparison of normalized current densities (*I*_2-APB_/*I*_CAP_) in HEK293T cells expressing WT, rTRPV1-G563S, or rTRPV1-R557K at ±60 mV (*I*_2-APB_/*I*_CAP_ for WT: 0.84 ± 0.06 at +60 mV and 0.91 ± 0.08 at −60 mV, *n* = 13; G563S: 0.03 ± 0.004 at +60 mV and 0.02 ± 0.004 at −60 mV, *n* = 8; R557K: 0.02 ± 0.001 at +60 mV and 0.02 ± 0.002 at −60mV, *n* = 5). Holding potentials were −60 mV with ramp-pulses (−100 to +100 mV, 300 ms) applied every 3 s. Data represent means ± SEM. Statistical analysis was performed using two-sample *t*-test, ***p < 0.001.

On the other hand, we unexpectedly observed rTRPV1 activation by 3 mM menthol (Fig. 3b), although we could not determine EC_50_ values of menthol-evoked activation for rTRPV1 since solutions with high menthol concentrations could not be prepared. As expected, rTRPV1-G563S and rTRPV1-R557K lost menthol-evoked activation, although capsaicin (1 μM) sensitivity was not affected (Fig. 3b–d and Supplementary Fig. 6a). However, reduction of capsaicin-evoked currents after washout occurred very slowly for the two mutants compared to WT-rTRPV1. A comparison of the current densities for WT, rTRPV1-G563S and rTRPV1-R557K 30 s after capsaicin washout showed that the two mutants had significantly larger current densities than WT (Supplementary Fig. 6c), indicating the involvement of these two residues in channel gating. We next examined the effects of camphor and 2-APB since their effects on mTRPV3 differed between G573 and R567 (Figs. 1 and 2). Interestingly, neither camphor nor 2-APB activated rTRPV1-G563S or rTRPV1-R557K (Fig. 4).

### Importance of positive charge at R567 for activation of mTRPV3 by camphor

**Fig. 5:**
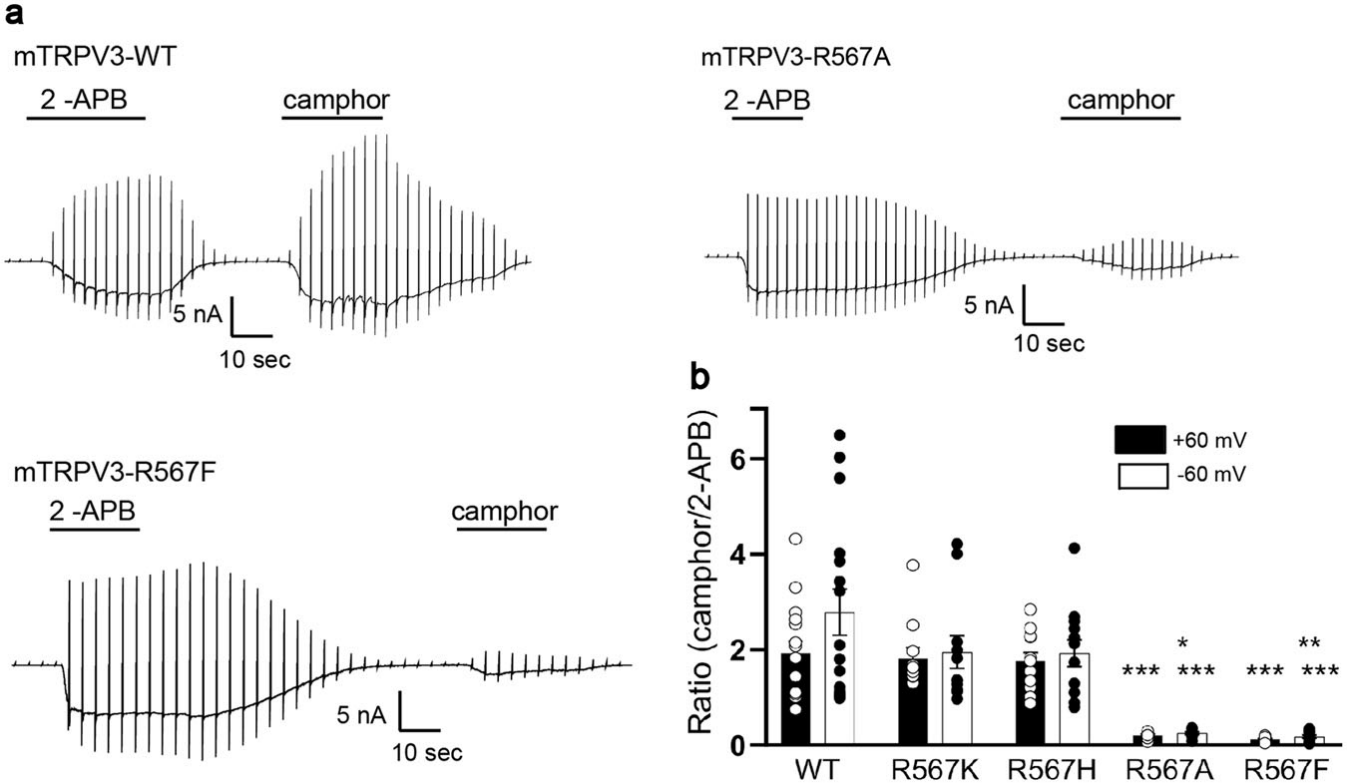
Positive charge of R567 is essential for camphor-evoked activation of mouse TRPV3. **a** Representative current traces for HEK293T cells expressing WT, mTRPV3-R567A, or mTRPV3-R567F in response to 2-APB (300 μM) treatment followed by camphor (10 mM) stimulation. **b** Comparison of normalized current densities (*I*_camphor_/*I*_2-APB_) in HEK293T cells expressing WT, mTRPV3-R567K, mTRPV3-R567H, mTRPV3-R567A, or mTRPV3-R567F at ±60 mV (WT: 1.93 ± 0.25 at +60 mV and 2.79 ± 0.48 at −60 mV, *n* = 16; R567K: 1.82 ± 0.22 at +60 mV and 1.95 ± 0.34 at −60 mV, *n* = 11; R567H: 1.77 ± 0.17 at +60 mV and 1.92 ± 0.28 at −60 mV, *n* = 12; R567A: 0.21 ± 0.02 at +60 mV and 0.25 ± 0.03 at −60 mV, *n* = 9; R567F: 0.13 ± 0.01 at −60 mV and 0.18 ± 0.03 at −60 mV, *n* = 12). Holding potentials were −60 mV with ramp-pulses (−100 to +100 mV, 300 ms) applied every 3 s. Data represent means ± SEM. Statistical analysis was performed by one-way ANOVA, *p < 0.05 (R567A vs. R567K, R567H at −60 mV), **p < 0.01 (R567F vs. R567K, R567H at −60 mV) and ***p < 0.001 (R567A vs. WT, R567K, R567H at +60 mV; R567F vs. WT, R567K, R567H at +60 mV; R567A vs. WT at −60 mV; R567F vs. WT at −60 mV).

The above data indicated that the involvement of glycine and arginine in monoterpene-evoked activation differs between mTRPV3 and rTRPV1. Due to the structural similarity of menthol and camphor as monoterpenes, we explored whether the charge at R567 is important for the camphor action by comparing the response of WT, R567K, R567H, R567A and R567F to camphor. We first confirmed that R567F was not sensitive to menthol (Supplementary Fig. 7). Moreover, since the response to 2-APB was not affected for the R567 mutants, we compared the ratio of camphor-activated currents to 2-APB-activated currents. Camphor-evoked mTRPV3 activation was markedly reduced for R567A and R567F (Fig. 5), confirming the importance of the positive charge of R567 for activation of mTRPV3.

### Involvement of phospholipids in monoterpene-induced activation of rTRPV1, but not mTRPV3

Recent structures of TRP channels have been instrumental in understanding how diverse ligands, including 2-APB, menthol, and others act on these channels and suggested that the pocket that binds these ligands also contains native lipid molecules. To understand the effect of specific lipid molecules on camphor-evoked currents, we depleted the specific lipid types from the membrane and then examined the modulation of channel functions by ligands　using two chemicals, the specific PI4 kinase inhibitor phenylarsine oxide (PAO)^35^ and wortmannin, which inhibits both PI3 and PI4　kinases with different concentration dependence^36,37^. We observed no difference in camphor-activated mTRPV3 currents in the presence and absence of either PAO (100 μM) or wortmannin (10 μM), whereas for rTRPV1 camphor-activated currents were significantly larger in the presence of PAO, but not wortmannin (Supplementary Fig. 8). Consistent with a previous report, PAO itself activated rTRPV1 (Supplementary Fig. 8b)^38^. Both chemicals are known to reduce PIP_2_ production, indicating that PIP_2_ has a larger role in activation of TRPV1 than TRPV3.

### Different role for the S4–S5 linker region in heat-evoked activation of TRPV1 and TRPV3

**Fig. 6:**
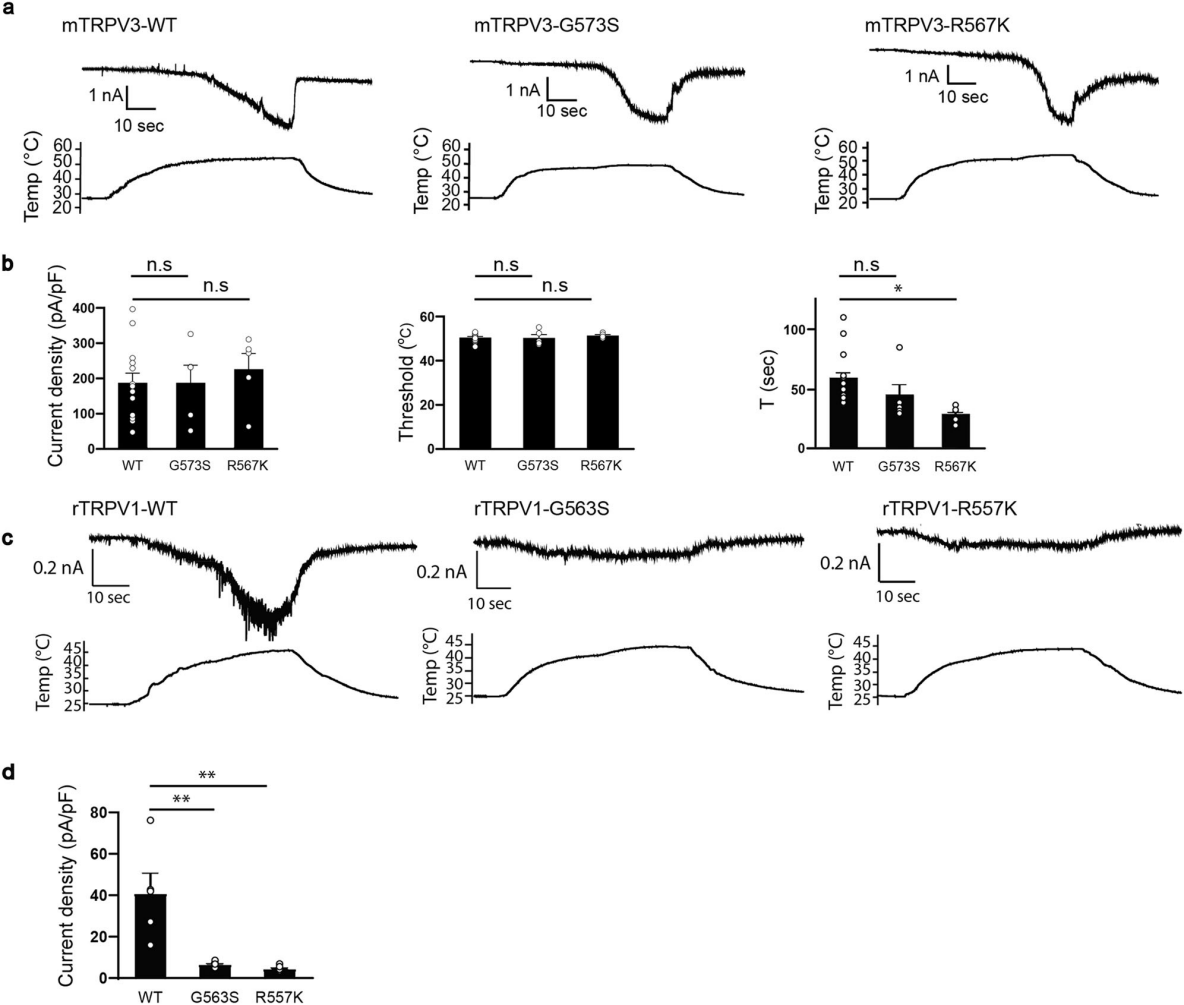
Heat-evoked currents for WT and mutated *mouse* TRPV3 and rat TRPV1. **a** Representative current (upper) and temperature (lower) traces for HEK293T cells expressing WT, mTRPV3-G573S, or mTRPV3-R567K exposed to heat up to 53 °C. **b** Comparison of heat-evoked current densities (left) (WT: 183.5 ± 26.1 pA/pF; G573S: 183.6 ± 49.5 pA/pF; R567K: 211.9 ± 44.1 pA/pF), temperature thresholds (middle) (WT: 50.3 ± 0.6 °C, *n* = 14; G573S: 50.2 ± 1.5 °C, *n* = 5; R567K: 51.2 ± 0.4 °C), *n* = 5, and time to current peak (WT: 58.2 ± 5.2 s, *n* = 14; G573S: 44.2 ± 9.4 s, *n* = 5; R567K: 28 ± 2.8 s, *n* = 5) in HEK293T cells expressing WT, mTRPV3-G573S, or mTRPV3-R567K at −60 mV. **c** Representative current (upper) and temperature (lower) traces for HEK293T cells expressing WT, rTRPV1-G563S, or rTRPV1-R557K exposed to heat up to 45 °C. **d** Comparison of heat-evoked current densities for HEK293T cells expressing WT, rTRPV1-G563S, or rTRPV1-R557K at −60 mV. Holding potentials were −60 mV (WT: 40.8 ± 10.1 pA/pF, *n* = 5; G563S: 6.5 ± 0.6 pA/pF, *n* = 6; R557K: 4.5 ± 0.6 pA/pF, *n* = 6). Data represent means ± SEM. Statistical analysis was performed by one-way ANOVA, *p < 0.05, and **p < 0.01.

Notably, temperature sensitivity is a hallmark of TRPV1 and TRPV3 channel function^4,39,40^. Mutagenesis or deletion studies indicated several regions important for heat-evoked activation of TRP channels. The heat-sensitive regions of TRP channels are located at the N-terminal ankyrin repeat domains in TRPA1 (ref. ^41^), a membrane-proximal N-terminal segment in TRPV1, TRPV2, and TRPV3 (refs. ^5,42^), the C terminus of TRPV1 (refs. ^43–45^), and the pore domain of TRPV1 and TRPV3 (refs. ^46–49^.). Residues that could be involved in temperature sensing may be scattered throughout the receptors rather than a defined temperature-sensing domain^50^. We thus considered whether glycine and arginine, which are involved in monoterpene-evoked activation, are also involved in heat-evoked activation. We first examined temperature-evoked activation of mTRPV3-G573S and mTRPV3-R567K in HEK293T cells by heating bath solution from 25 °C to 50−53 °C. Similar to WT mTRPV3, large inward currents were observed for both mTRPV3-G573S and mTRPV3-R567K without changes in temperature thresholds (Fig. 6a, b and Supplementary Fig. 9). However, activation of mTRPV3-G573S and mTRPV3-R567K by heat stimulus appeared more rapid than for WT. Therefore, we compared the activation time course of the heat-evoked currents. The times between the current rise and the peak were shorter for mTRPV3-G573S and mTRPV3-R567K than for WT, although only the difference between WT and mTRPV3-R567K was statistically significant (Fig. 6b). These data suggested that these residues are involved in the heat-evoked activation of mTRPV3. On the other hand, rTRPV1-G563S and rTRPV1-R557K did not exhibit heat-evoked activation (Fig. 6c, d), whereas capsaicin sensitivity was not affected (Fig. 3d and Supplementary Fig. 6a). These data indicated that glycine and arginine in S4–S5 of mTRPV3 and rTRPV1 have a different involvement in heat-evoked activation, and the finding that G563 and R557 are involved in heat-evoked activation of rTRPV1 is consistent with a previous report^34^.

### Stable binding of menthol and camphor by *mouse* TRPV3

**Fig. 7:**
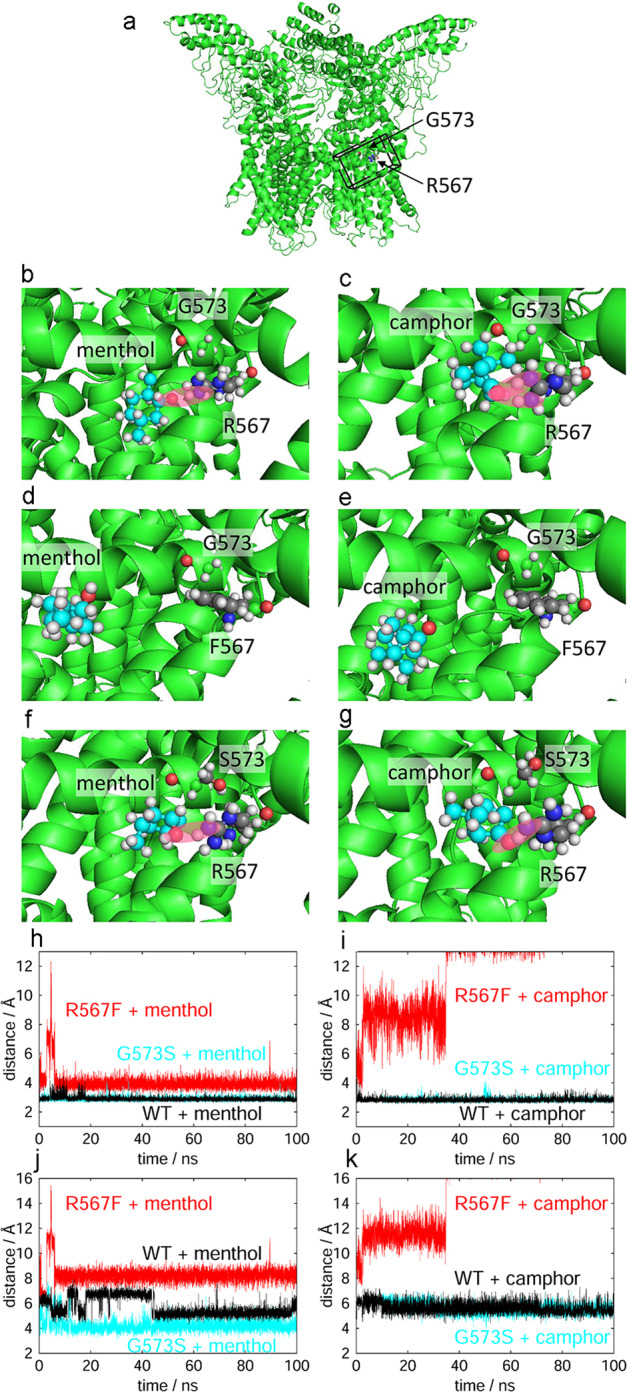
Molecular dynamics simulation confirming a stable bond between monoterpenes and R567. Representative snapshots of **a** the whole WT TRPV3 system, **b** menthol in WT TRPV3, **c** camphor in WT TRPV3, **d** menthol in mTRPV3-G573/R567F, **e** camphor in mTRPV3-G573/R567F, **f** menthol and mTRPV3-G573S/R567, **g** camphor and mTRPV3-G573S/R567. Black frame in **a** represents the area we focus in **b**–**g**. Pink highlights indicate hydrogen bonds between monoterpenes and R567. Time series of distance between **h** menthol and R567/F567 and **i** camphor and R567/F567. Time series of distance between **j** menthol and G573/S573 and **k** camphor and G573/S573.

To examine the structural features of the interaction of TRPV3 with menthol and camphor, we performed MD simulations (Fig. 7a–k). The oxygen atoms of menthol and camphor forms hydrogen bonds with amino groups (−NH_2_) of R567 (Fig. 7b, c, f, g) that promote stable binding of menthol and camphor to this residue in mTRPV3. On the other hand, these hydrogen bonds cannot form for the R567F mutant (Fig. 7d, e), since the phenylalanine does not have an amino group. Thus, menthol and camphor are not fixed in the R567F mutant and are sometimes distant from F567. To observe this phenomenon more clearly, we calculated a time series for the distances between menthol/camphor and R567/F567 (Fig. 7h, i). The distance was calculated as the shortest distance between the heavy atoms (except hydrogen) of menthol/camphor and those of R567/F567. This distance fluctuated slightly around 3 Å for WT and G573S, but ranged between 3 and more than 10 Å for R567F mutant. These results show that both menthol and camphor stably bind to R567 via hydrogen bonding in WT and G573S, but in the R567F mutant the binding states fluctuate. We also calculated the distances between menthol/camphor and G573/S573, as shown in Fig. 7j, k. In the R567F mutant, the distance from G573 to the monoterpenes also fluctuated in the wide range as in the distance from F567. In WT and G573S, the fluctuation of the distance from G573/S573 was much smaller than that in R567F, but slightly more than the distance from R567. This fact indicates that the monoterpenes are bound to R567, but not to G573/S573. In Fig. 7j, the distance in G573S fluctuated less than that in WT. This is because the mutation of G573 to serine caused the steric overlap with W692 and the binding pocket became smaller to eliminate this overlap, meaning that G573 is not involved in ligand binding, but affects the size of the ligand-binding pocket.

## Discussion

To date, little is known about how different monoterpenes are sensed by TRPV3 and the sites at which they act are also unclear. Investigation of the actions of monoterpene agonists on TRPV3 will provide clues to clarify the structural basis of activation, which could lead to increased understanding of the role of TRPV3 in physiological function and in disease. We can also exploit the promiscuous actions of monoterpenes toward several TRP channels as an important research tool to characterize the physiological function and biophysical properties of ion channels. Several monoterpene compounds such as menthol and camphor, and the synthetic small, hydrophobic compound 2-APB all activate TRPV1 and TRPV3, even though these two channels share only about 39% amino acid sequence identity^21,51,52^. In the present work, we identified the putative shared amino acids that are involved in menthol, camphor, and 2-APB-evoked activation in mTRPV3 and rTRPV1 and these residues were further confirmed in MD simulations. Our results showed that the conserved glycine and arginine residues in the S4–S5 linker of mTRPV3 (G573, R567) and rTRPV1 (G563, R557) are required for menthol- and camphor-evoked activation (Supplementary Table 2). Although we did not examine 2-APB binding to the glycine and arginine in TRPV3 in MD simulations, 2-APB actions involving these two residues are consistent with previous reports^16,51,53^.

As previously reported, several amino acids function as binding sites for menthol in mTRPM8 (Y745 (S1), R842 (S4), and Y1005 (TRP domain))^10,23^. Among these, Y745 and R842 are conserved in TRPV1 and TRPV3. Y745, R842, and Y1005 are proximal in the TRPM8 structure, but do not lie close to one another in the TRPV1 and TRPV3 structures (Supplementary Fig. 1). PIP_2_, a positive modulator of the TRPM8 channel, can bind to the pocket formed by the pre-S1 elbow, S1 of one subunit, and S5 in the neighboring subunit^9^. On the other hand, it was shown that menthol has a bimodal action on mTRPA1 via a mechanism that involves S876 and T877 in S4–S5 linker/S5 region^3^. These results suggest that the binding pocket and mechanistic action of menthol toward TRPM8 would be distinguishable from that for other TRP channels, and that the S4–S5 linker/S5 region is critical for the action of menthol in TRPV1, TRPV3, and TRPA1, but not TRPM8.

In this study we revealed the activation of rTRPV1 by menthol, which had characteristics similar to that seen for camphor-mediated activation of rTRPV1. Xu et al.^21^ showed that camphor has strong desensitizing activity of rTRPV1, and suggested that the combination of desensitization of rTRPV1 and inhibition of rTRPA1 could provide a new explanation for the pain-relieving properties of menthol. These actions could be parallel to the analgesic property of menthol, since high concentrations of menthol inhibit both mammalian TRPA1 and TRPV1 (refs. ^23,26^). Moreover, total outcomes in terms of whole-body sensation would be very interesting to examine. As we previously reported, menthol activates TRPM8, TRPV3, TRPA1, and TRPV1 and inhibits TRPV1 and TRPA1 (at high concentrations for the *mouse* clone). We think that total outcomes would be determined by which channel has the most prominent activation. Menthol activates TRPM8 most effectively at relatively low concentrations, explaining why exposure to menthol typically induces a cooling sensation.

In our study we found some differences in the actions of menthol, camphor, and 2-APB between rTRPV1 and mTRPV3. The single point mutations G563S and R557K in rTRPV1 eliminated sensitivity to menthol, camphor, and 2-APB (Figs. 3 and 4), whereas the G573S and R567K mutations exhibited different responses from those seen for mTRPV3. G573S lost sensitivity to all three compounds, and the sensitivity of R567 to menthol was affected, but that for 2-APB was not. Although the structures of camphor and menthol are similar, the positive charge at R567 was necessary for activation of mTRPV3 by camphor (Fig. 5). These results could be explained in part by the smaller size of camphor relative to menthol that requires the positive charge to promote an effective fit in the binding pocket formed by G573 and R567.

Remarkably, the G573S mutation of human TRPV3 corresponding to *mouse* TRPV3-G573S in our study is associated with Olmsted syndrome, which is characterized by bilateral mutilating palmoplantar keratoderma and periodic keratotic plaques accompanied by severe itching^54,55^. This mutation in mTRPV3 was reported to show large basal currents in the absence of agonists and no responses to 2-APB and camphor in either HEK293 cells or *Xenopus* oocytes^29,56^. Meanwhile, in this study basal currents before stimulation were not larger than those for WT-expressing HEK293T cells. We also did not see 2-APB and camphor sensitivity for this mutant (Fig. 1) or differences in heat sensitivity between mTRPV3-WT and G573S (Fig. 6). The reason for the apparent difference in the basal channel activities between this study and previous studies is unclear. However, the cryo-EM-based structure indicates that G573 in the S4–S5 linker lies at the junction with the lower part of S4, S5, the TRP domain, and the lower part of S6 (ref. ^57^). This structural feature raises the possibility that mTRPV3-G573 is involved in channel gating in concert with W692, which is in the vicinity of G573 and reported to cause large basal currents^29,56^. However, the mechanisms responsible for this high basal activity remain unknown.

The mutants G563S and R557K essentially lost the heat sensitivity of rTRPV1, while G573S and R567K exhibited similar heat-evoked responses in WT mTPRV3 with a small difference in activation kinetics, indicating that the involvement of these two corresponding residues in heat sensitivity differs between mTRPV3 and rTRPV1 (Fig. 6). No clear evidence for the mechanisms of heat-evoked activation of TRP channels is available, although multiple studies involving mutant analyses or cryo-EM studies showed that several regions are involved in the heat sensitivity of TRPV1 and TRPV3 (refs. ^32,43–46,58^). Our results indicated that the activation mechanisms of heat and chemical stimulation of TRPV3 differ, but could have somehow converged in the sensitivity of TRPV1 to some chemicals such as monoterpenes, suggesting that mechanisms for heat sensitivity of TRPV3 and TRPV1 are different. These results could clarify the mechanisms of heat-evoked activation of thermosensitive TRP channels. Singh et al. structurally identified two conformational steps for the heat-evoked activation of TRPV3: a strongly temperature-dependent first step (sensitization) and a weakly temperature-dependent second step (channel opening)^59^. In both steps, heat stimuli induce a conformational change in the ankyrin repeat domain that consequently promotes a structural change in the transmembrane domain. Moreover, in both steps TRPV3 shows strong gating-associated changes in annular lipid occupancy wherein the first density is sandwiched between the extracellular half of S4 in one subunit and the pore domain of the adjacent subunit. The second density lies in a pocket formed by the intracellular half of the S1–S4 domain and the C-terminal portion of the TRP domain^59^. Interestingly, a recent structure involving lipid nanodisc-reconstitution of TRPV3 reveals a lipid–protein interaction in TRPV3 and a bound lipid stabilized the selectivity filter of the pore in the narrow state since the lipid displacement in this region might be involved in the structural transitions from close-π to open state during TRPV3 activation^60^. Although the type and exact role of these putative lipids in thermo-sensitization was unclear due to the limited resolution of the structures, the authors highlighted an important role of lipids in heat sensing by TRPV3 through its interactions with the surrounding membrane lipids.

Several studies suggested that TRPV1 is intrinsically heat sensitive^61^, and that manipulating the cholesterol composition in native membranes likely does not play a critical role in temperature sensing^62^. However, the structure of TRPV1 in nanodiscs revealed the presence of a phosphatidylinositol lipid between the S4 of one subunit and S5 and S6 of an adjacent subunit. This finding suggested that heat may open the channel through displacement of resident phosphatidylinositides in this S4–S5 linker region. In this earlier study, R557 of rTRPV1 binds to PIP_2_, which is consistent with our results. In addition, Wen et al.^63^ observed in an MD simulation heat-activated conformation changes in several key domains including the S4–S5 linker of TRPV1, indicating the importance of this region for heat-evoked activation. They also found that heat-evoked expansion of the TRPV1 pore is greater at 72 °C than 60 °C, and such expansion was detected in the open structure of TRPV1, thus supporting a putative role for the S4–S5 linker in TRPV1 gating. Although the residues found in this study substantially reduced the heat-evoked activation of rTRPV1, whether they contribute directly to temperature sensing or have other roles in allosteric coupling remains unclear.

Given that both G573 and R567 may be involved in the binding of monoterpenes in mTRPV3, we performed MD simulations focusing on these residues (Fig. 7). We confirmed that the tight binding of monoterpenes to R567 was lost in the R567F mutant. Interestingly, however, we saw no significant changes in the distance between glycine or serine of mTRPV3 and monoterpenes even though monoterpene-induced currents were substantially reduced for G573S (Fig. 1). These data suggest that G573 is not involved in ligand binding, but rather in channel gating. On the other hand, the amplitudes and temperature thresholds for heat-evoked currents were not different between WT and the mutants (G573S and R567K), although the time to peak of heat-evoked R567K currents was significantly shorter compared with WT (Fig. 6), suggesting that R567 is involved in both heat-evoked channel gating and ligand binding. Glycine at 573 in mTRPV3 is well-conserved in TRP channels, suggesting the importance of this residue in channel function. Indeed, in rTRPV1 the corresponding residue G563 was found to be involved in multiple functions including channel activation by small hydrophobic compounds, heat-evoked activation, and desensitization of capsaicin-activated currents^34^. Taken together, we concluded that G573 is involved in mTRPV3 gating while R567 is involved in both ligand binding and heat-evoked channel activation. The role of G573 in channel gating could be invoked downstream of ligand binding at R567. This possibility would be consistent with a report that G573 interacts with residues in the TRP box that modulates channel gating^64^.

The S4–S5 linker mediates gating motions in many ion channels with six transmembrane domains^65^. For voltage-gated potassium (Kv) and sodium (Nav) channels, conformational changes in S4 are coupled to movement of the S4–S5 linker that promotes opening of the activation gate. Several studies indicated the importance of the S4–S5 linker in ligand binding of TRPV1. Gao et al. suggested that vanilloid agonists function by displacing the resident phosphatidylinositol lipid in the pocket formed by the S4 and S4–S5 linker. Using Rosetta-based molecular docking, Yang et al.^66^ observed a common structural mechanism underlying vallinoid-evoked activation of TRPV1 where the ligand serves as molecular “glue” that bridges the S4–S5 linker to the S1–S4 domain to induce channel opening. In addition, the authors found that, upon binding, capsaicin initiates a conformational wave that propagates through the S4–S5 linker toward the S6 bundle to open the restriction site in the selectivity filter, consequently inducing conformational rearrangements of the selectivity filter that eventually lead to channel opening^67^. Since there are putative lipids located in the S4–S5 interface in TRPV1 and TRPV3 (refs. ^15,16,57,60^), we hypothesize that monoterpenes displace the lipid in the S4–S5 interface pocket and in turn displace the S4–S5 linker away from the central axis to mediate S5 and S6 movement, leading to channel activation. However, the exact mechanism may differ between TRPV1 and TRPV3 since there are some differences in these channels such as the kind of lipid, the regulation of lipid, and the properties of the S6 helix related to channel gating^15,16,53,60,68^. However, whether there is a rearrangement of the S1–S4 bundle upon ligand binding that contributes to channel opening is unclear, particularly since monoterpenes are generally smaller than vanilloids. Therefore, mimicking the action of vanilloid in several other channels would be difficult^66,69^.

Menthol and camphor, as mentioned above, are small and hydrophobic (Log *p* value of 3.4 and 2.74 (refs. ^28,70^), respectively). Several previous studies showed that the physico-chemical characteristics of lipid bilayer membranes such as membrane fluidity and thickness can be modified by menthol and other related monoterpenes (refs. ^27,28,71^). TRPM8 requires PIP_2_ for activation and can be activated by PIP_2_ alone^9–13^. Interestingly, beyond the positive effects of PIP_2_ in menthol-evoked activation of TRPM8 and TRPV3 (ref. ^72^), previous studies also reported the location of putative lipids in several regions including the S4–S5 linker in TRPV1, TRPV3, and TRPV6 (refs. ^15,16,57^). For example, the pocket at the S4–S5 interface that binds to an activating lipid in TRPV6 (the density observed in this pocket may represent the natural lipid agonist in TRPV6-binding sites), and the exchange between phosphoinositides and activator/inhibitor in this vanilloid-binding pocket, could allosterically regulate TRPV1. Indeed, 2-APB is proposed to induce TRPV3 activation and inhibit TRPV6 by modulating interactions with bound lipids^16^. Thus, we suggest that monoterpenes could modulate the activity of several TRP channels including TRPM8, TRPV1, and TRPV3 by manipulating interactions with bound lipids, particularly since monoterpenes can act on several channels and the binding pocket locations overlapped with those for lipids. Regarding the involvement of PIP_2_ in channel activation by monoterpenes, PAO increased camphor-evoked rTRPV1 currents, but not mTRPV3 (Supplementary Fig. 8). This result supports the involvement of PIP_2_ in TRPV1 activity^61^. On the other hand, the negative results for mTRPV3 suggest a less important role for PIP_2_ in TRPV3 activity, despite a report showing that TRPV3 activity is potentiated by PIP_2_ with PAO^72^. Even with these data, we cannot exclude the possibility that other lipids are involved in modulating TRPV3 and TRPV1 activity. In any case, activation or inhibition of TRP channel activity by monoterpenes and the intensity with which these agents act could depend on the regulatory roles of lipids for TRP channel gating and on what kinds of lipid, such as PIP_2_ and cholesterol, bind to the channels. However, we cannot rule out that both changes in the physico-chemical properties of the lipid bilayer and direct binding of hydrophobic compounds to ion channels explain the actions of menthol and other monoterpenes on the functional properties of TRP channels and other ion channels.

## Methods

### Construction of mutant *mouse* TRPV1, TRPV3, or TRPM8

TRPV3 or TRPM8 point mutants were constructed using a PrimeSTAR mutagenesis Basal kit according to the manufacturer’s recommendations (Takara Bio Inc., Shiga, Japan). TRPV1 point mutants were constructed using PrimeSTAR GXL DNA polymerase (Takara Bio Inc., Shiga, Japan). Point mutations were introduced by PCR using *rat TRPV1*-pcDNA3, *mouse TRPV3*-pcDNA3, and *mouse TRPM8*-pcDNA5/FRT as templates with oligonucleotide primers (Supplementary Table 1) containing the intended mutations. The amplified PCR products were transformed into *Escherichia coli* pcDNA3 vectors containing *rat TRPV1* or *mouse TRPV3*, or pcDNA5/FRT vectors containing *mouse TRPM8* that were then purified using standard procedures. The entire *rat TRPV1*, *mouse TRPV3*, and *mouse TRPM8* coding sequences were determined to confirm that only the intended mutations were introduced.

### Cell culture

Human embryonic kidney‐derived 293T (HEK293T) cells were maintained at 37 °C and 5% CO_2_ in Dulbecco’s modified Eagle’s Medium (WAKO Pure Chemical Industries, Osaka, Japan) containing 10% fetal bovine serum (Biowest SAS, Nuaillé, France), 100 units ml^−1^ penicillin (Invitrogen Corp.), 100 μg ml^−1^ streptomycin (Invitrogen Corp.), and 2 mm GlutaMAX (Invitrogen Corp.). For patch‐clamp recordings, 1 μg *mouse TRPM8* in pcDNA5/FRT, *rat TRPV1* or *mouse TRPV3* in pcDNA3 vector and 0.1 μg pGreen Lantern 1 cDNA were transfected to HEK293T cells cultured in 35 mm dishes using Lipofectamine Plus Reagent (Invitrogen Corp.). After incubating for 3–4 h, the cells were reseeded on coverslips and further incubated at 33 °C for *mouse TRPV3* or 37 °C for *rat* TRPV1 or *mouse* TRPM8 in 5% CO_2_. Patch‐clamp recordings were performed 1 day after transfection.

### Chemicals

*l*-menthol, capsaicin, 2-aminoethoxydiphenyl borate, phenylarsine oxide (PAO), and wortmannin were purchased from Sigma-Aldrich (St. Louis, MO, USE) and camphor was purchased from WAKO chemicals (Tokyo, Japan). 2-APB, PAO, and wortmannin were dissolved in DMSO to make 1 M, 300 mM, and 20 mM stock solutions. Camphor and *l*-menthol were dissolved in ethanol to make 3 M stock solutions that were diluted to the desired final concentration with bath solution.

### Electrophysiology

For whole‐cell experiments, the experimental solutions were bath solution: 140 mM NaCl, 5 mM KCl, 2 mM MgCl_2_, 5 mM EGTA, 10 mM HEPES, and 10 mM glucose at pH 7.4 adjusted with NaOH; pipette solution: 140 mM CsCl, 5 mM EGTA, and 10 mM HEPES at pH 7.4 adjusted with CsOH. Data from whole‐cell voltage‐clamp recordings were acquired at 10 kHz throughout the experiments and filtered at 5 kHz for analysis (Axon 200B amplifier with pCLAMP software; Axon Instruments, Foster City, CA, USA). The membrane potential was clamped at −60 mV. Series resistance and membrane capacitance were compensated.

All experiments were performed at 25 °C unless otherwise stated. Heat stimulation was induced by increasing the bath temperature using a pre‐heated solution warmed in an inline heater (1 °C s^−1^, with a maximum of 55 °C). The temperature was monitored using a thermocouple (TC‐344; Warner Instruments, Hamden, CT, USA) placed within 100 μm of the patch‐clamped cell. The heat stimulation was stopped upon confirming that *rat* TRPV1 or *mouse* TRPV3 currents were desensitized or inactivated. Temperature profiles and Arrhenius plots for the data from whole‐cell voltage‐clamp recordings were calculated using Origin 8.5 software (OriginLab, Northampton, MA, USA). The absolute current values were plotted on a log scale against the reciprocal of the absolute temperature (*T*) (Arrhenius plot), and the temperature threshold for channel activation was determined by the temperature that caused a change in the slope. For current density analysis of TRPV3 channels, the peak currents induced by heat or chemical stimulation were measured and presented as pA pF^−1^.

### Molecular dynamics simulation

To confirm stable binding of monoterpenes, we performed molecular dynamics (MD) simulations of TRPV3 with a *l*-menthol molecule and a camphor molecule. The PDB structure (ID: 6DVW) was used as the initial structure for TRPV3. Because the N-terminal (residues 1–114) and C-terminal (residues 759–791) residues are absent in the PDB structure, the N terminus (residue 115) and C terminus (residue 758) were blocked with an acetyl group and a *N*-methyl group, respectively. Docking conformations of the *l*-menthol and camphor molecules with TRPV3 were obtained using the docking program AutoDock^73^. The N- and C-terminal residues (up to residue 391 and after residue 720) were removed in our MD simulations. We also performed MD simulations for the TRPV3 mutant R567F with both *l*-menthol and camphor. The initial conformation of the R567F mutant was prepared by replacing the arginine residue (R567) with a phenylalanine residue. In addition, MD simulations for G573S were performed with the monoterpenes. The initial conformation was prepared as follows. Residue 573 was replaced from a glycine residue to a serine residue, as with R567F. Because the serine residue was sterically overlapped with W692, potential energy minimization was carried out to remove the overlap. After the minimization, the docking program AutoDock was utilized again to obtain docking conformations of the monoterpenes with G573S.

The electrostatic charges of atoms in the *l*-menthol and camphor molecules were determined using restrained electrostatic potential fits^71^. Quantum chemical calculations were performed using the Gaussian16 program^74^. Structure optimization and electrostatic potential calculations were carried out using the Hartree–Fock level with a 6-31G(d) basis set. For parameters other than atomic electrostatic charges, General Amber force field parameters^75^ were used.

The MD simulations were performed using the Generalized-Ensemble Molecular Biophysics program developed by one of the authors (H.O.). This program has been used for several proteins and peptides^76,77^. The AMBER parm14SB force field^78^ was used for TRPV3. A cubic unit cell having a side length of 200 Å with periodic boundary conditions was used. The electrostatic potential was calculated with the particle-mesh Ewald method^79^. The cut-off distance for the Lennard–Jones potential was 12 Å. The Nosé–Hoover thermostat^80,81^ was used to control the temperature at 310 K and a multiple-time-step method^82^ was used. The time step was set as ∆*t* = 0.5 fs and ∆*t* = 2.0 fs for bonding and non-bonding interactions, respectively. To maintain the atomic structure of TRPV3 in a vacuum, the N, C_α_, and C atoms of residues involved in α-helix structures were restrained with a harmonic potential^83^. Each MD simulation was performed for 100 ns.

### Statistics and reproducibility

Data for the patch-clamp experiments were obtained from at least three independent transfections. Data are presented as the mean ± SEM. Statistical analysis was performed with Origin 8.5 software (OriginLab, Haverhill, MA, USA). Significant changes were identified using a two-sample *t*-test, or one-way ANOVA followed by a Bonferroni post hoc test with *p* < 0.05 considered as statistically significant (*p* values: *<0.05, **<0.01, ***<0.001). EC_50_ values was determined using Origin 8.5 software.

### Reporting summary

Further information on research design is available in the Nature Research Reporting Summary linked to this article.

## Supplementary information

Supplementary Information

Description of Additional Supplementary Files

Supplementary Data 1

Supplementary Data 2

Supplementary Movie 1

Supplementary Movie 2

Reporting Summary

## Data Availability

All data and materials used in the analysis are available in the main text, Figures and Supplementary Figures and Tables. EC_50_ values are available in the end of Supplementary Data 1 and 2 for *rat* TRPV1 and *mouse* TRPV3, respectively. The demonstrations of MD simulations are available in Supplementary Movies 1 and 2. Primer sequences and a summary of mutant responses are available in Supplementary Tables 1 and 2, respectively. Other data or information that support the findings of this study are available from the corresponding author M.T (tominaga@nips.ac.jp) upon request.
