# Supplementary material for: Structural basis for promiscuous action of monoterpenes on TRP channels
Source: Commun Biol. 2021 Mar 5;4:293. doi: 10.1038/s42003-021-01776-0 (PMC7935860; doi:10.1038/s42003-021-01776-0)
Supplement: Supplementary file 1 — Supplementary Information [file 42003_2021_1776_MOESM1_ESM.pdf]

Structural basis for promiscuous action of monoterpenes on TRP channels

Structural basis for promiscuous action of monoterpenes on TRP channels

Thi Hong Dung Nguyen[1,2,3], Satoru G. Itoh[4,5,6], Hisashi Okumura[4,5,6], Makoto Tominaga*[1,2,3]

[1]Department of Physiological Sciences, SOKENDAI, Okazaki, Japan

[2]Division of Cell Signaling, National Institute for Physiological Sciences, Okazaki, Japan

[3]Thermal Biology Group, Exploratory Research Center on Life and Living Systems (ExCELLS), Okazaki, Japan

[4]Biomolecular Dynamics Simulation Group, ExCELLS, Okazaki, Japan

[5]Research Center for Computational Science, Institute for Molecular Science, Okazaki, Japan.

[6]Department of Structural Molecular Science, SOKENDAI, Okazaki, Japan

* Corresponding Author: Makoto Tominaga, Division of Cell Signaling, National Institute for Physiological Sciences, National Institutes of Natural Sciences, Higashiyama 5-1, Myodaiji, Okazaki, Aichi 444-8787 JAPAN,
Phone: +81-564-59-5286, Fax: +81-564-59-5285, E-mail: tominaga@nips.ac.jp,

Supplementary Figure 1. Ribbon diagrams of mouse TRPM8, mouse TRPV3, and rat TRPV1 structures.

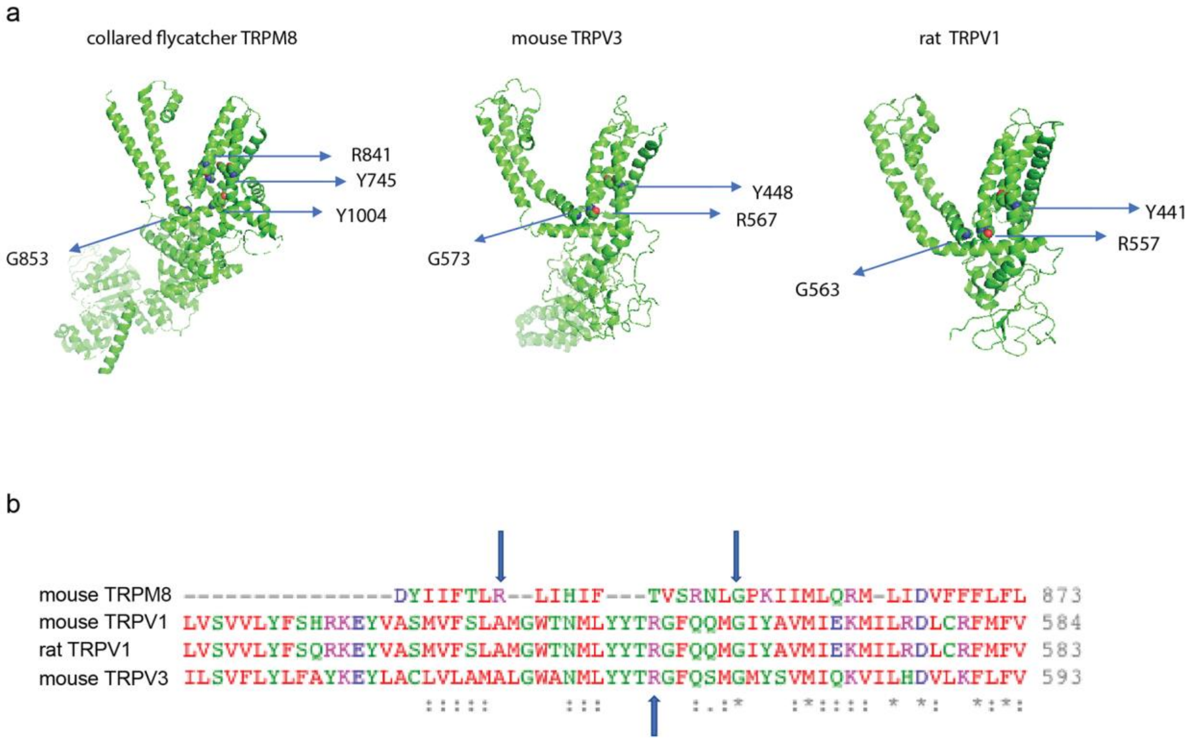

(a) Three amino acids (tyrosine, Y; glycine, G; arginine, R) are shown in each ribbon model.

(b) Alignment of S4-S5 linker sequences for *mouse* TRPM8 (PDB ID: 6BPQ), *rat* TRPV1(PDB ID: 3J5P), and *mouse* TRPV3 (PDB ID: 6DVW). Conserved arginine and glycine residues are shown by arrows.

Supplementary Figure 2. Involvement of mouse TRPM8 residues Y745 (S1) and R842 (S4) in the agonistic effect of menthol.

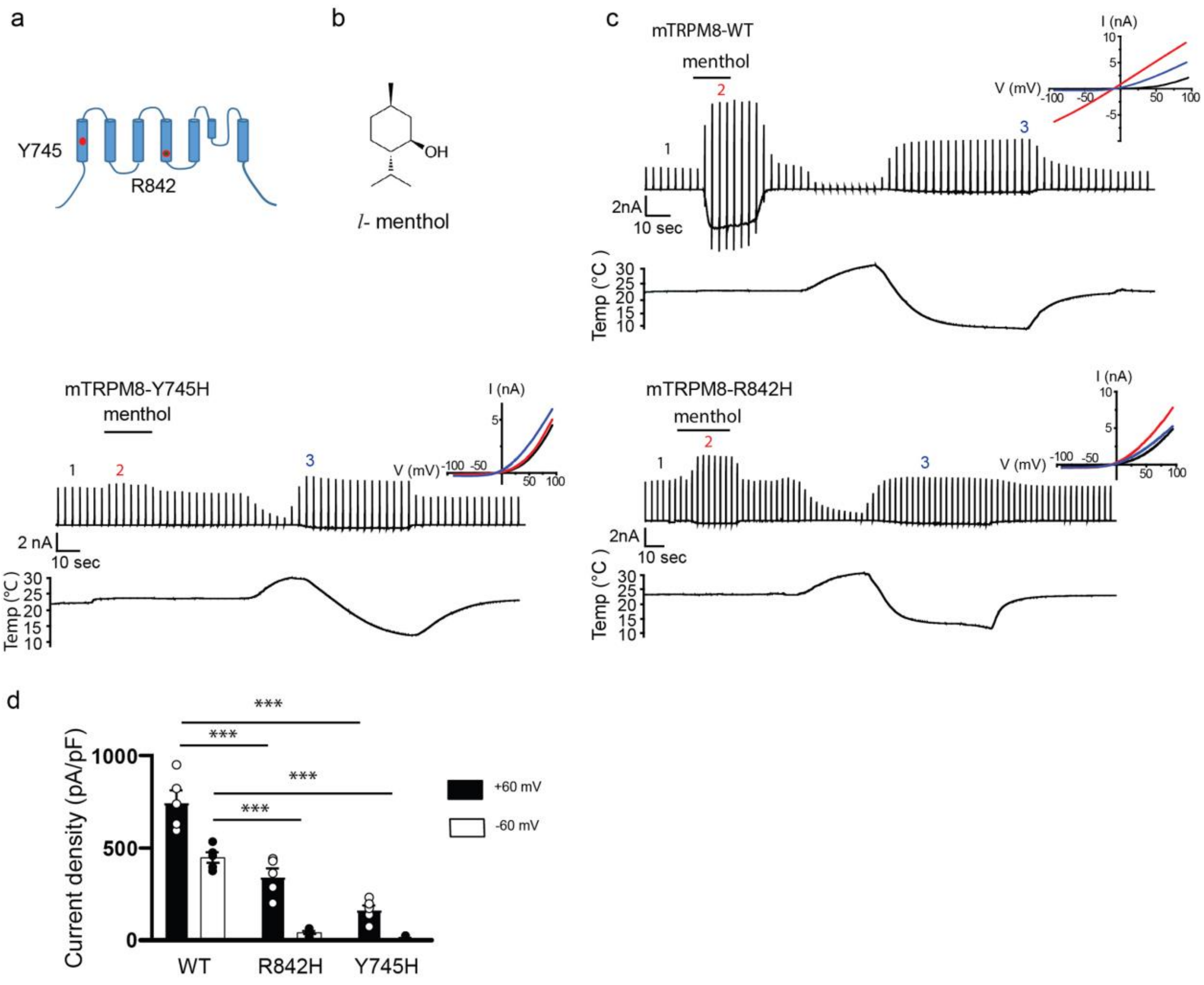

(a) Location of amino acids involved in menthol binding by *mouse* TRPM8 (mTRPM8). (b) Structure of *l*-menthol. (c) Representative current traces for HEK293T cells expressing wild type (WT), mTRPM8-Y745H, or mTRPM8-R842H. Insets indicate current-voltage curves obtained at different time points (1, 2, 3) shown in each trace. Holding potentials were -60mV with ramp-pulses (-100 ~ +100mV, 300ms) applied every 3 sec. (d) Comparison of current densities at ±60 mV in HEK293T cells expressing WT, Y745H, or R842H mTRPM8. (WT: 749.4 ± 64.8 pA/pF at +60mV and 449.4 ± 28.6 at -60mV, n = 5, R842H: 345.5 ± 45.2 pA/pF at +60mV and 42.5 ± 7.6 pA/pF at -60mV, n = 5, Y745H: 166.4 ± 22.3 pA/pF at +60mV and 10.3 ± 3.0 pA/pF at -60mV, n = 6). Data represent means ± S.E.M. Statistical analysis was performed by One way ANOVA followed by a Bonferoni test, *** $p < 0.001$.

Supplementary Figure 3. Comparison of current densities for HEK293T cells expressing *mouse* TRPV3 WT or Y448H activated by menthol, camphor, or 2-APB.

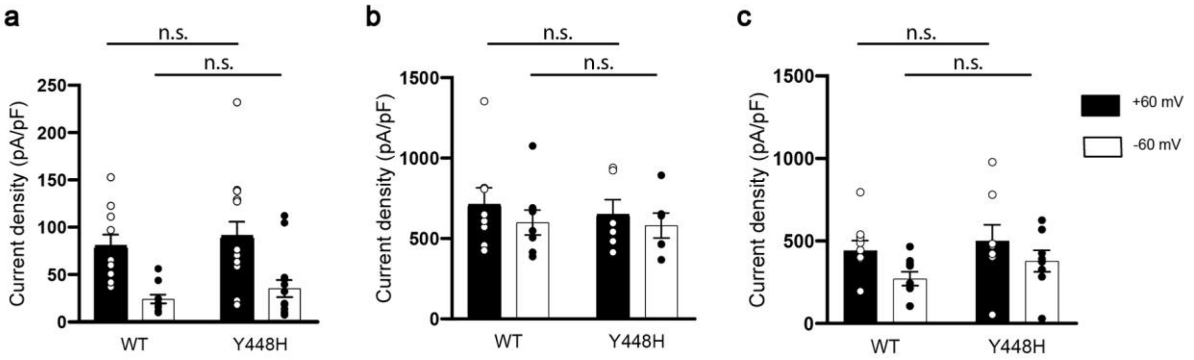

Comparison of current densities following treatment of HEK293T cells expressing WT or mTRPV3-Y448H with **(a)** menthol (3 mM) (WT: 80.1 ± 3.2 pA/pF at +60mV and 24.1 ± 4.7 at -60mV, n = 10, Y448H: 90.7 ± 15.2 pA/pF at +60mV and 35.2 ± 9.0 pA/F at -60mV, n = 14), **(b)** camphor (10 mM) (WT: 711.3 ± 104.6 pA/pF at +60mV and 599.7 ± 77.8 pA/pF at -60mV, n = 8, Y448H: 649.7 ± 92.6 pA/pF at +60mV and 580.9 ± 77.3 pA/pF at -60mV, n = 6), or **(c)** 2-APB (300 μM) (WT: 441.4 ± 61.1 pA/pF at +60mV and 270.8 ± 41.9 pA/pF at -60mV, n = 9, Y448H: 501.1 ± 91.8 pA/pF at +60mV and 377.9 ± 64.6 pA/pF at -60mV, n = 8). Holding potentials were -60mV. Data represent means ± S.E.M. Statistical analysis was performed by two samples *t*-test.

Supplementary Figure 4. Response of HEK293T cells expressing WT or R567K of *mouse* TRPV3 to menthol, camphor, or 2-APB.

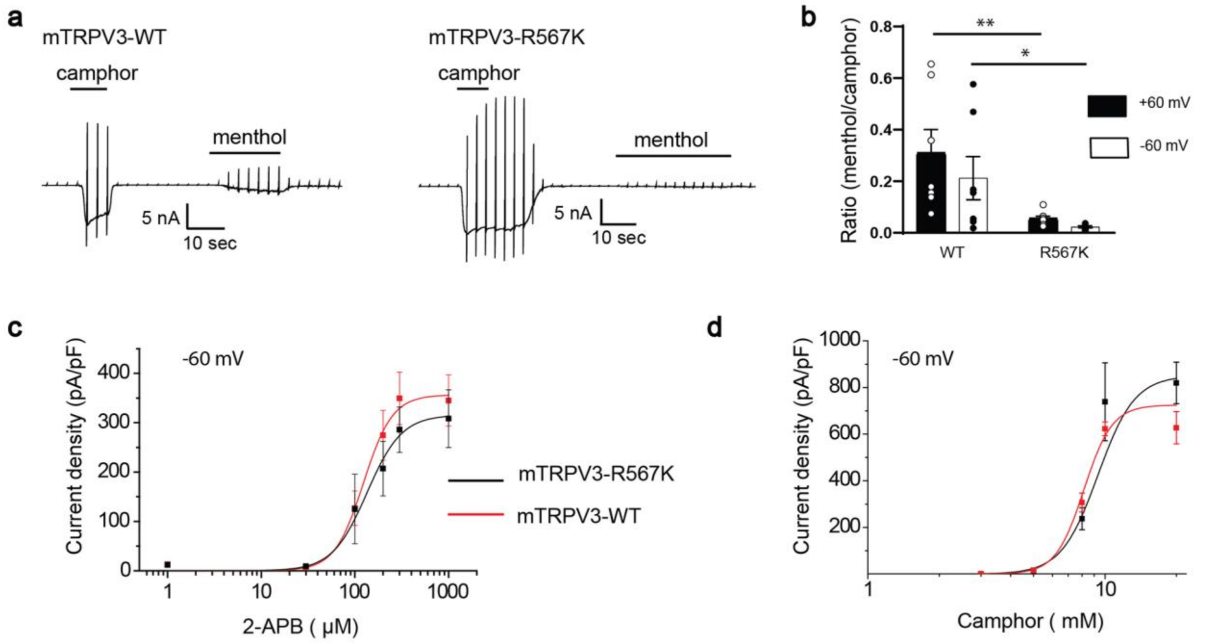

(a) Responses to camphor (10 mM, first) and menthol (3 mM, second) by HEK293T cells expressing WT or mTRPV3-R567K. Holding potentials were -60mV with ramp-pulses (-100 ~ +100mV, 300ms) applied every 3 sec. (b) Comparison of normalized current densities ($I_{menthol}/I_{camphor}$) in HEK293T cells expressing WT or mTRPV3-R567K at ±60mV (WT, 0.31 ± 0.09 at +60mV and 0.2 ± 0.08 at -60mV, n = 7, R567K: 0.06 ± 0.01 at +60mV and 0.02 ± 0.003 at +60mV, n = 9). Data represent means ± S.E.M. Statistical analysis was performed by two samples *t*-test, * $p < 0.05$ and ** $p < 0.01$. (c) Dose-dependent curves of 2-APB-activated currents for WT (black) and mTRPV3-R567K (red) at -60 mV. EC_{50} values are 134.4 ± 81 and 123.5 ± 33 µM for WT and R567K, respectively. Hill co-efficient values are 2.8 and 2.3 for WT and mTRPV3-R567K, respectively. Curves were fit with the data (n = 4-14). (d) Dose-dependent curves of camphor-activated currents for WT (black) and mTRPV3-R567K (red) at -60 mV. EC_{50} values are 9.3 ± 0.7 and 8.1 ± 0.5 mM for WT and R567K, respectively. Hill co-efficient values are 5.6 and 7.3 for WT and mTRPV3-R567K, respectively. Curves were fit with the data (n = 4-14).

Supplementary Figure 5. Comparison of densities of WT or F569H *mouse* TRPV3 currents activated by menthol and camphor.

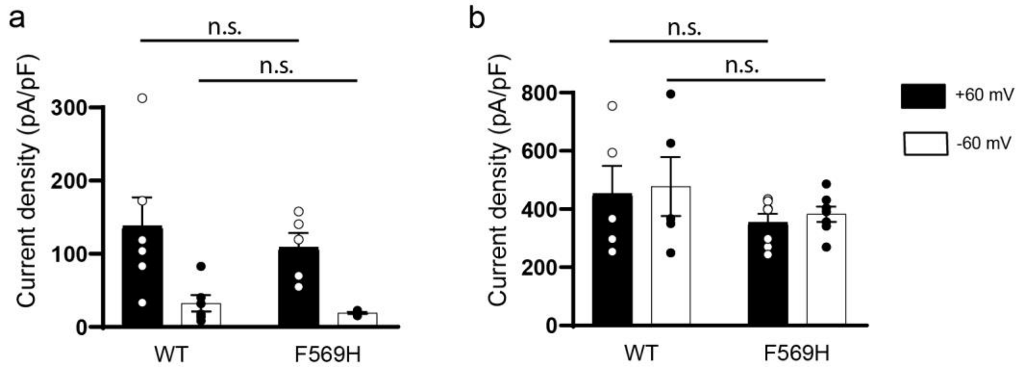

(a) Comparison of densities of currents activated by menthol (3 mM) (WT: 137.4 ± 39.7 pA/pF at +60 mV and 36.1± 11.7 pA/pF at -60mV; F569H: 108.5 ± 18.1 pA/pF at +60mV and 20.2 ± 1.2 pA/pF at -60mV) in HEK293T cells. (b) Comparison of densities of currents activated by camphor (10 mM) (WT: 453.3 ± 87.2 pA/pF at +60mV and 477.6 ± 92.1 pA/pF at +60mV; F569H: 353.4 ± 30.4 pA/pF at +60mV and 382.9 ± 26.2 pA/pF at -60mV) in HEK293T cells. Data represent means ± S.E.M. (n = 5). Statistical analysis was performed by two samples *t*-test.

Supplementary Figure 6. Capsaicin-evoked responses of WT, G563S, R557K and Y441H in *rat* TRP1.

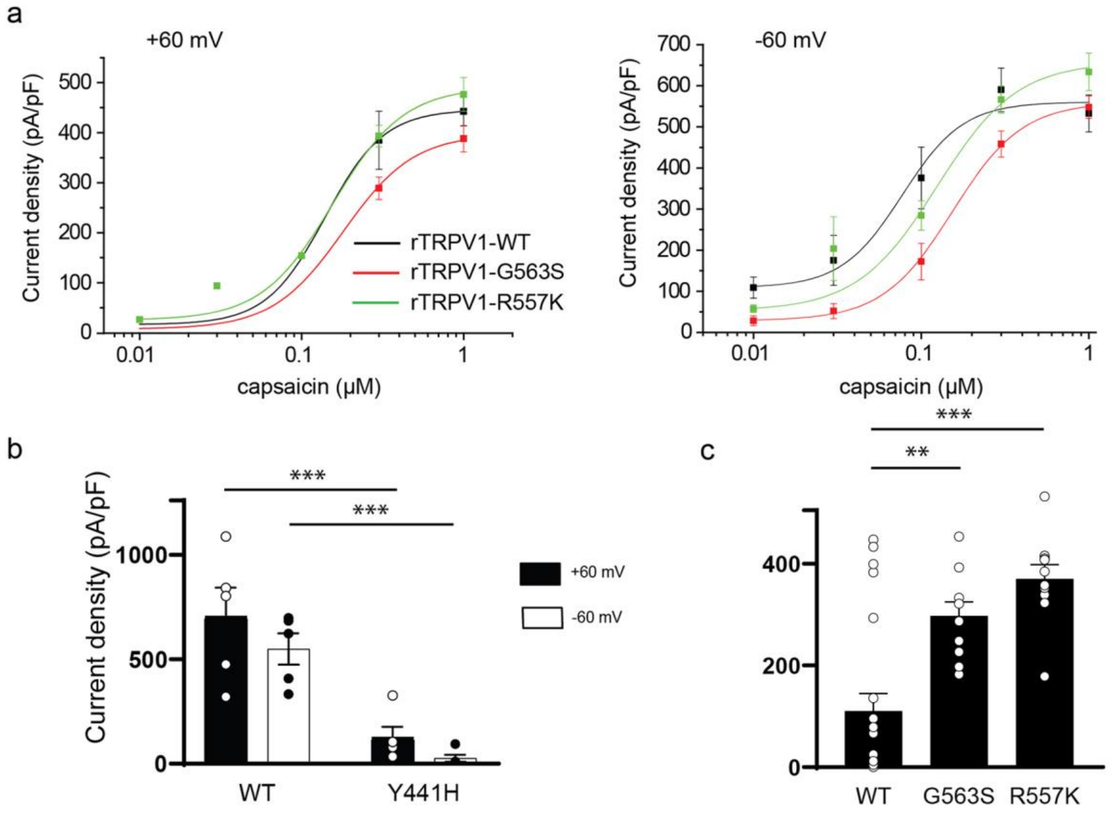

(a) Dose-dependent curves for capsaicin-activated currents of WT (black), rTRPV1-G563S (red) and rTRPV1-R557K (green) at +60 mV (left) and -60 mV (right) in HEK293T cells (n=4-22). EC_{50} values are 140 ± 4 and 77 ± 30 nM at +60 and -60 mV, respectively for WT; 180 ± 30 and 154 ± 10 nM at +60 and -60 mV, respectively for G563S; 156 ± 30 and 124 ± 30 nM at +60 and -60 mV respectively for R557K. Hill co-efficient values are 2.4 and 2.4 at +60 and -60 mV, respectively, for WT; 2.0 and 2.0 at +60 and -60 mV, respectively for rTRPV1-G563S; 1.8 and 2.0 at +60 and -60 mV, respectively for rTRPV1-R557K. (b) Comparison of densities of currents activated by capsaicin (1 μM) in HEK293T cells expressing WT or rTRPV1-Y441H at ±60mV (WT: 706.0 ± 137.0 pA/pF at +60mV and 549.3 ± 75.0 pA/pF at -60mV, Y441H: 125.7 ± 47.1 pA/pF at + 60mV and 26.6 ± 15.4 pA/pF at -60mV). Data represent means ± S.E.M. (n = 5). Statistical analysis was performed by two samples *t*-test, *** $p < 0.001$. (c) Comparison of the extent of current reduction 30 sec after capsaicin washout at -60 mV (WT: 110 ± 34.4 pA/pF, n = 22, G563S: 296.5 ± 27.3, n = 14, R557K: 368.7 ± 28.2, n = 11). Statistical analysis was performed by One way ANOVA followed by a Bonferoni test, ** $p < 0.01$ and *** $p < 0.001$.

Supplementary Figure 7. Responses of WT, R567H, R567K, or R567F *mouse* TRPV3 to 2-APB, camphor, or menthol.

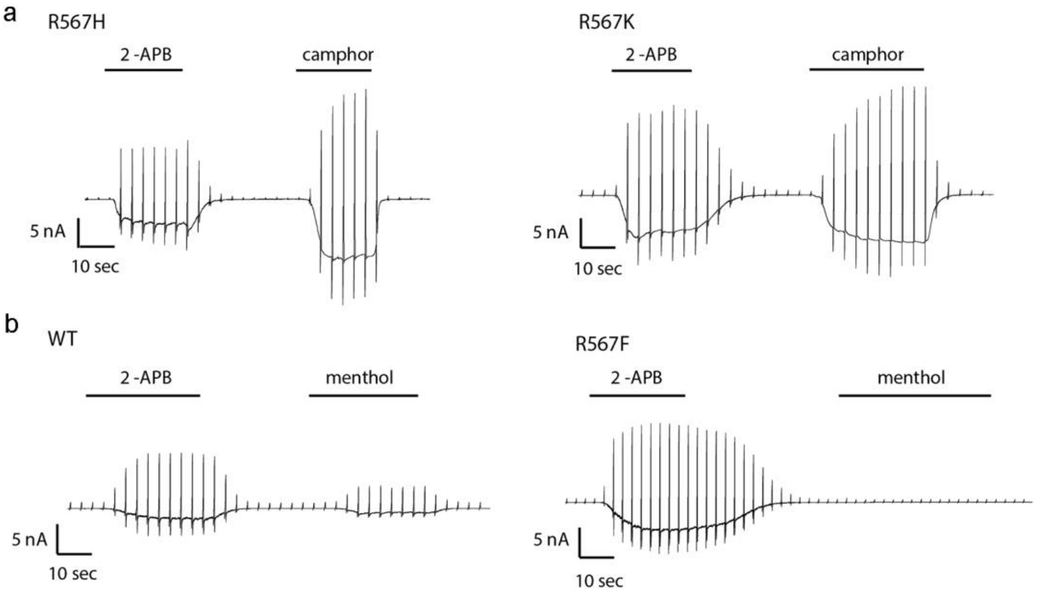

(a) Representative traces of currents elicited in response to 2-APB (300 μM, first) and camphor (10 mM, second) in HEK293T cells expressing mTRPV3-R567H or mTRPV3-R567K. (b) Representative traces of currents elicited in response to 2-APB (300 μM, first) and menthol (3 mM, second) in HEK293T cells expressing WT or mTRPV3-R567F. Holding potentials were -60mV with ramp-pulses (-100 ~ +100mV, 300ms) applied every 3 sec.

Supplementary Figure 8. Responses of WT *mouse* TRPV3 or rat TRPV1 to camphor in the presence of phenylarsine oxide or wortmannin.

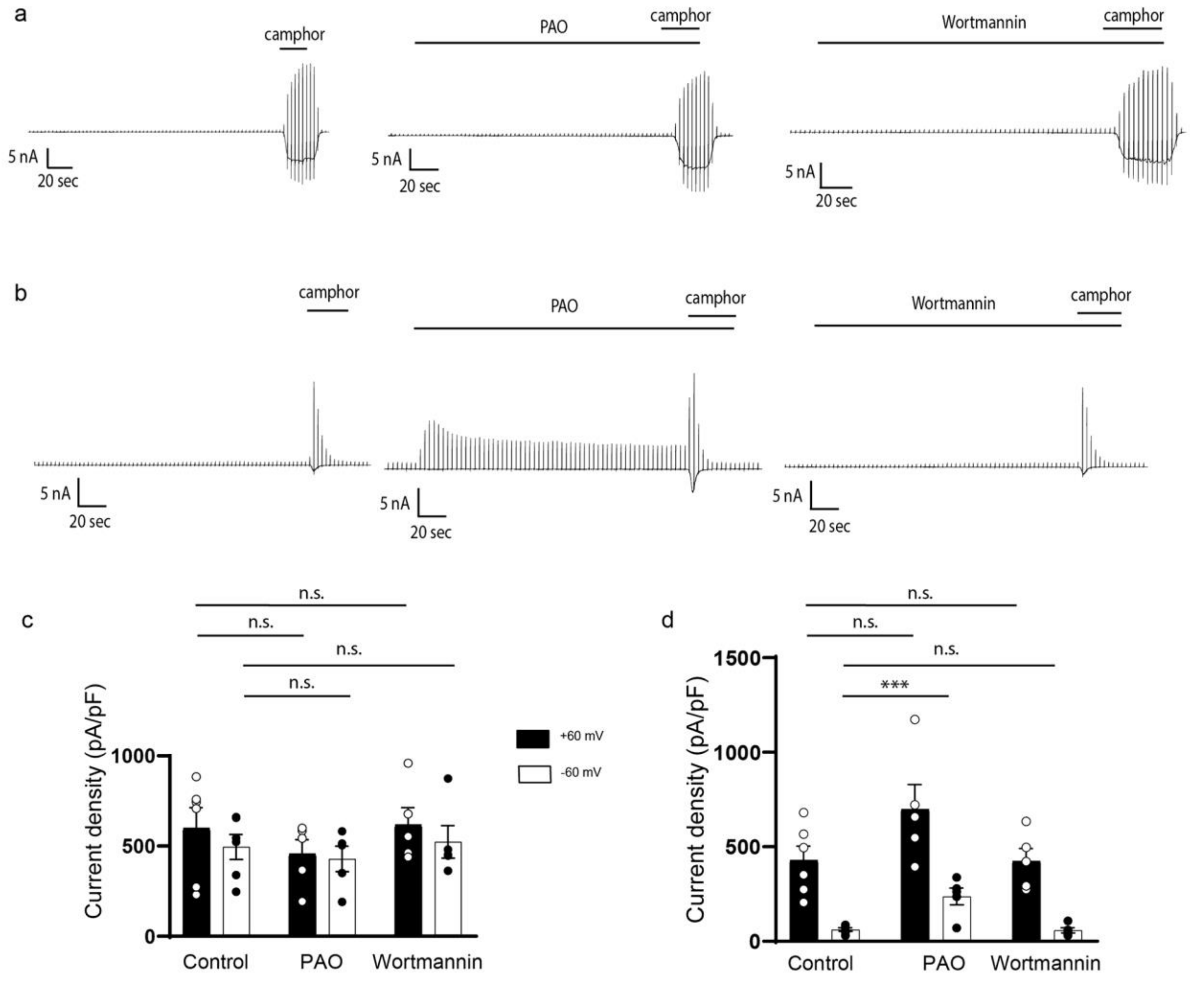

(a) Representative traces of the camphor (8 mM)-activated mTRPV3 currents in the absence (left) or presence of phenylarsine oxide (PAO, 100 µM, middle) or wortmannin (10 µM, right). (b) Representative traces of the camphor (10 mM)-activated rTRPV1 currents in the absence (left) or presence of PAO (100 µM, middle) or wortmannin (10 µM, right). (c) Comparison of densities of mTRPV3 currents activated by camphor in the absence (560 ± 113 pA/pF at +60 mV and 495.1 ± 69 pA/pF at -60mV, n = 6) or presence of PAO (457.4 ± 78.1 pA/pF at +60mV and 428.4 ± 70.6 pA/pF at -60mV, n = 5) or wortmannin (618.7 ± 94.8 pA/pF at +60mV and 523.5 ± 90 pA/pF, n = 5). (d) Comparison of densities of rTRPV1 currents activated by camphor in the absence (699 ± 120 pA/pF at +60mV and 237.5 ± 41 pA/pF at -60mV, n = 6) or presence of PAO (492.2 ± 74.4 pA/pF at +60mV and 62.2 37.3 ± 9.6 pF/pA at -60 mV, n= 5) or wortmannin (424.2 ± 66.6 pA/pF and 58.4 ± 13.5 pA/pF at -60mV, n = 5). Data represent means ± S.E.M. Statistical analysis was performed by One way ANOVA followed by a Bonferoni test, *** $p < 0.001$.

Supplementary Figure 9. Arrhenius plots of *mouse* TRPV3 currents activated by heat stimulation.

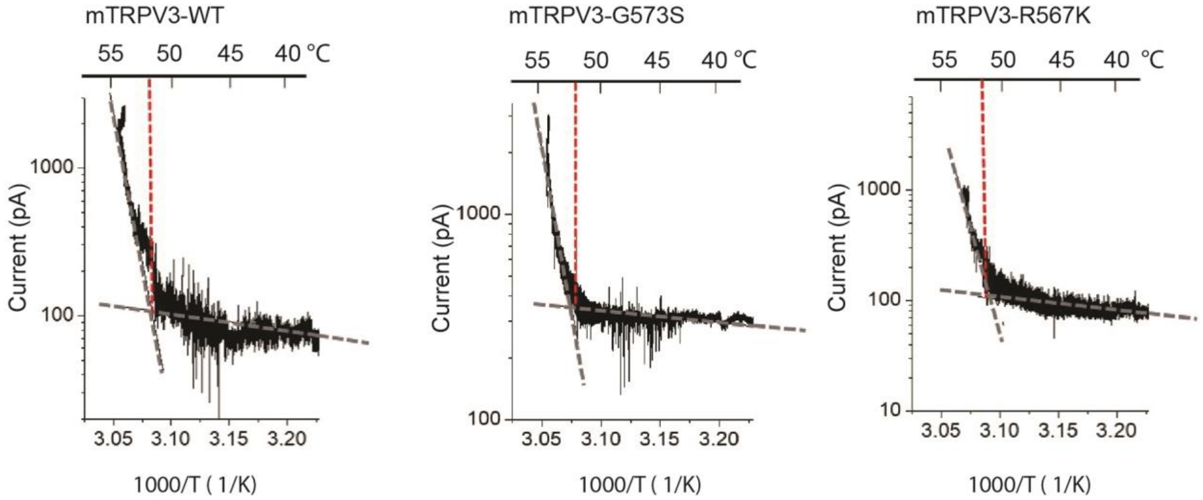

Representative Arrhenius plots from currents activated by heat stimulation of HEK293T cells expressing WT, mTRPV3-G573S or mTRPV3-R567K at -60 mV. Gray dotted lines indicate linear fits of the plots before and after heat-evoked activation, and red dotted lines indicate temperature thresholds at the intersection of the gray dotted lines.

Supplementary Table 1.

Primers for making mutants

	Mutation	Sense primer (5' -> 3')	Antisense primer (5'->3')
mouse TRPM8	Y745H	GTCTTCcAtATCGCCTTCCTCCTGCT	GGCGATaTgGAAGACCACGTTCCAGG
	R842H	ACGCTAcacCTCATCCACATTTTCAC	GATGAGgtgTAGCGTGAATATAATGT
rat TRPV1	Y441H	GCAgTGGACGAAGAAGTTGAAGTAGAAG	GCAgTGGACGAAGAAGTTGAAGTAGAAG
	R557K	TATACCaaAGGATTCCAGCAGATGGGC	GAATCCttTGGTATAGTAGAGCATGTTGG
	G563S	CAG ATGaGCATCTATGCTGTCATGATT	ATGCTCcACTATACCCGAGGATTCCA
mouse TRPV3	Y448H	TGCTTCcATTTCTTCTACAACATCACC	GAAGAAATgGAAGCAGAAGGACAAGAA
	R567K	TACACGAaAGGCTTCCAGTCTATGGG	GAAGCCTtTCGTGTAGTAGAGCATGTT
	R567H	TACACGcacGGCTTCCAGTCTATGGG	GAAGCCgtgCGTGTAGTAGAGCATGTT
	R567A	TACACGgcAGGCTTCCAGTCTATGGG	GAAGCCTgcCGTGTAGTAGAGCATGTT
	R567F	TACACGttcGGCTTCCAGTCTATGGG	GAAGCCgaaCGTGTAGTAGAGCATGTT
	G573S	TCTATGaGCATGTACAGCGTCATGATCCA	GTACATGCtCATAGACTGGAAGCCTC
	F569H	GAGAGGccACCAGTCTATGGGCATGTA	AGACTGgtGGCCTCTCGTGTAGTAGAGC

Supplementary Table 2.

Summary of the mutant responses

mTRPV3	G573S	- Loss of activation by all three ligands (camphor, menthol, 2-APB)<br>- Faster kinetics of heat-evoked activation
	R567K	- Reduced menthol-evoked activation; camphor, 2-APB effects unchanged<br>- Faster kinetics of heat-evoked activation
	R567H	- Reduced menthol-evoked activation; camphor, 2-APB effects unchanged
	R567A and R567F	- Reduced menthol-evoked activation; reduced camphor-evoked activation; no change in 2-APB effects
rTRPV1	G563S	- Loss of menthol, camphor or 2-APB-evoked activation<br>- Capsaicin-evoked activation does not lead to current decay over time.<br>- Loss of heat-evoked activation
	R557K	- Loss of menthol, camphor or 2-APB-evoked activation.<br>- Capsaicin-evoked activation does not lead to current decay over time.<br>- Loss of heat-evoked activation
