# Supplementary material for: Structural basis for promiscuous action of monoterpenes on TRP channels
Source: Commun Biol. 2021 Mar 5;4:293. doi: 10.1038/s42003-021-01776-0 (PMC7935860; doi:10.1038/s42003-021-01776-0)
Supplement: Supplementary file 2 — Description of Additional Supplementary Files [file 42003_2021_1776_MOESM2_ESM.pdf]

Description of additional supplementary files

File name: Supplementary Data 1
Description: Source data for TRPV1 dose-dependent curves

File name: Supplementary Data 2
Description: Source data for TRPV3 dose-dependent curves

File name: Supplementary Movie 1
Description: Molecular Dynamics simulation trajectory of menthol in WT TRPV3, mTRPV3-R567F and mTRPV3-G573S

File name: Supplementary Movie 2
Description: Molecular Dynamics simulation trajectory of camphor in WT TRPV3, mTRPV3-R567F and mTRPV3-G573S
